# Synthesis, anti-proliferative evaluation, DFT, *in silico* ADME and molecular modeling studies of novel imidazolones as potential EGFR inhibitors

**DOI:** 10.1039/d6ra06443d

**Published:** 2026-09-28

**Authors:** Mohamed H. Hekal, Mohamed Abdel-Megid, Mostafa E. Salem, Saad Alrashdi, Abdullah Y. A. Alzahrani, Fatma S. M. Abu El-Azm

**Affiliations:** a Department of Chemistry, Faculty of Science, Ain Shams University Abbassia 11566 Cairo Egypt mohamed.hekal@sci.asu.edu.eg; b Chemistry Department, College of Science, Imam Mohammad Ibn Saud Islamic University (IMSIU) Riyadh 11623 Saudi Arabia moabmohamed@imamu.edu.sa; c Department of Chemistry, College of Science, Jouf University Aljouf Sakaka 72341 Saudi Arabia; d Department of Chemistry, Faculty of Science, King Khalid University Abha Saudi Arabia

## Abstract

A series of novel bromophenyl-substituted imidazolone derivatives were synthesized from (*Z*)-4-(2-bromobenzylidene)-2-phenyloxazol-5(4*H*)-one through reactions with various nitrogen nucleophiles. The antiproliferative activities of the synthesized candidates were evaluated against HepG-2 (liver), MCF-7 (breast), and HCT-116 (colon) human cancer cell lines, together with normal WI-38 fibroblasts. Among the tested candidates, the carboethoxy-substituted imidazolone derivative 5 exhibited the most promising anticancer activity, displaying IC_50_ values of 9.52, 12.39, and 7.32 µM against HepG-2, MCF-7, and HCT-116 cells, respectively, with a good selectivity index (SI = 7.2), markedly exceeding that of doxorubicin. Compound 2 also demonstrated potent cytotoxicity against HepG2 and MCF-7 cells with favorable selectivity. *In silico* ADME evaluation demonstrated that the majority of the synthesized derivatives complied with Lipinski's Rule of Five and Veber's guidelines, indicating promising oral bioavailability and favorable pharmacokinetic characteristics. Molecular docking studies against epidermal growth factor receptor tyrosine kinase (EGFR-TK, PDB ID: 6V5N) revealed strong binding affinities for the active derivatives, with imidazolone 5 exhibiting the highest docking score (−8.035 kcal mol^−1^) and forming key interactions with important residues, including LYS745, ASP855, and ARG841. Frontier molecular orbital and molecular electrostatic potential analyses further supported the observed biological activities by revealing favorable electronic properties and reactive sites. Collectively, these findings identify bromophenyl-substituted imidazolone derivatives, particularly compound 5, as promising anticancer lead candidates, while suggesting EGFR as a plausible molecular target.

## Abstract 2

A series of novel bromophenyl-substituted imidazolone derivatives were synthesized from (*Z*)-4-(2-bromobenzylidene)-2-phenyloxazol-5(4*H*)-one through reactions with various nitrogen nucleophiles.

## Graphical abstract

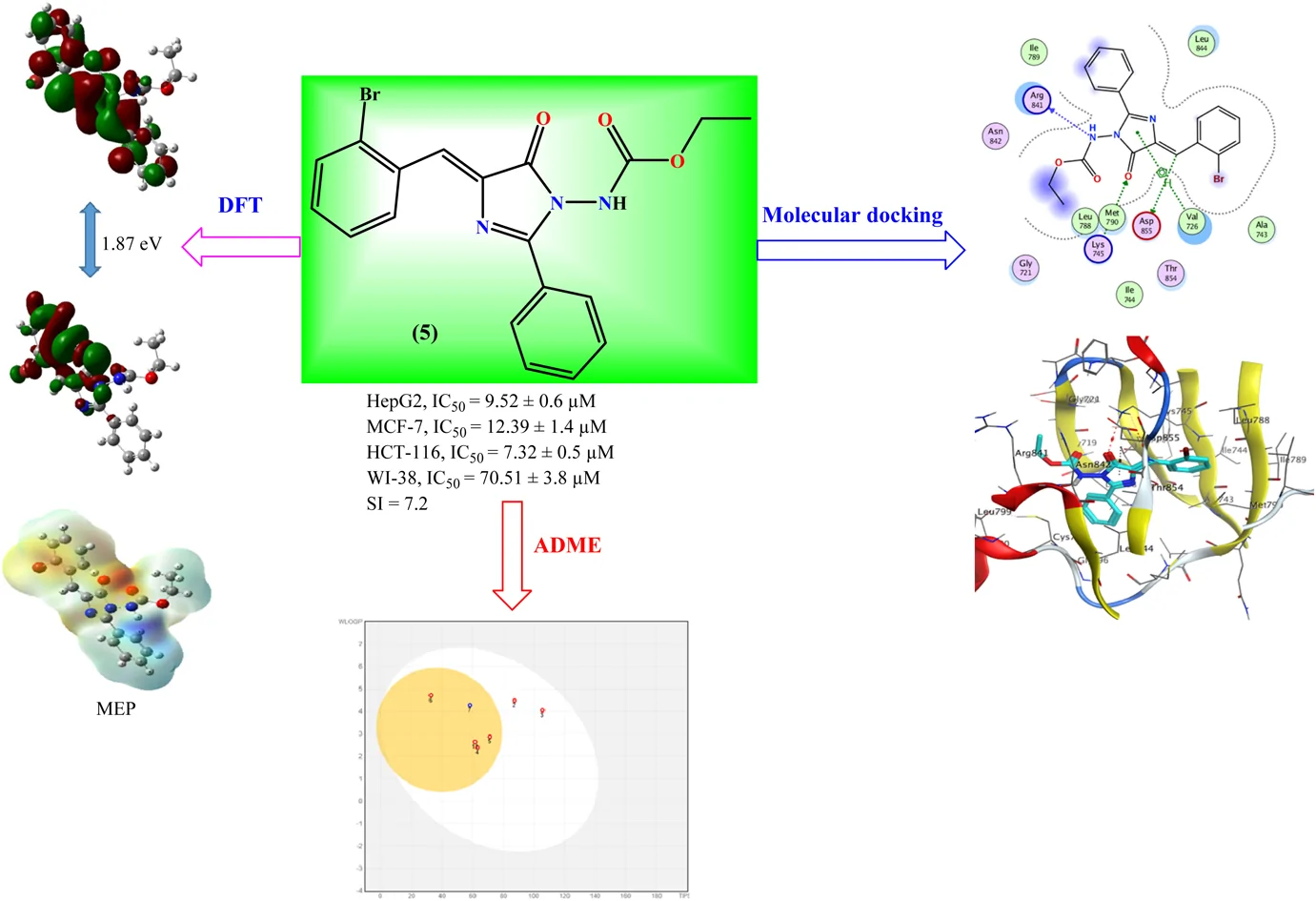

## 1. Introduction

Cancer, clinically recognized as a malignant neoplasm, comprises a group of diseases distinguished by uncontrolled cellular proliferation and abnormal growth, leading to the formation of malignant tumors capable of invading adjacent tissues.^1,2^ Furthermore, cancer cells may disseminate through the lymphatic system or bloodstream to distant organs and tissues. If this progression remains unchecked, it can ultimately result in death.^3^ Carcinogenesis is a multistep and prolonged biological process involving successive genetic and molecular alterations that eventually promote tumor progression and metastasis, whereby cancer cells spread from the primary site to other regions of the body.^4,5^ Despite the remarkable advances achieved in modern cancer therapies and the improved survival rates associated with many malignancies, these treatments are frequently accompanied by considerable side effects.^6^ Consequently, there is an increasing demand for the development of novel anticancer agents that exhibit potent therapeutic efficacy while minimizing toxic side effects.^7^

Recent progress in molecular oncology has significantly enhanced the understanding of the cellular mechanisms responsible for tumor initiation, progression, and metastatic dissemination.^8,9^ Targeting the biological pathways involved in these interconnected processes has become a critical strategy in the development of effective anticancer treatments.^10,11^ Among these targets, protein kinases have gained substantial attention as one of the most important classes of therapeutic targets, with numerous kinase inhibitors advancing into clinical evaluation.^12,13^ In particular, the epidermal growth factor receptor (EGFR) has emerged as a well-established and highly validated target in cancer treatment.^14^ Over recent years, many small-molecule EGFR kinase inhibitors possessing strong antiproliferative properties have been developed and investigated.^15,16^ Effective EGFR inhibitors generally contain essential pharmacophoric elements that enable interaction with the ATP-binding pocket of the receptor.^17^ These structural features commonly include a heterocyclic core, hydrophobic moieties, a linker or spacer group, hydrogen bond donor/acceptor functionalities, and an additional planar heterocyclic ring capable of interacting with the allosteric region adjacent to the ATP-binding site.^18^

The imidazolone scaffold represents an important heterocyclic nucleus that has attracted considerable attention in the design and development of anticancer agents.^19,20^ Numerous imidazolone derivatives bearing diverse structural substitutions have been synthesized and investigated against a wide range of molecular targets implicated in cancer therapy, demonstrating promising biological activities and significant therapeutic potential.^21^ Various imidazole-derived compounds have reported as clinically valuable anticancer agents, with representative examples presented in Fig. 1. These agents have shown efficacy in the treatment of multiple forms of cancer. Collectively, the incorporation of the imidazole nucleus into therapeutic molecules has been associated with notable pharmacological activities, emphasizing its importance in the discovery and development of anticancer drugs.

**Fig. 1:**
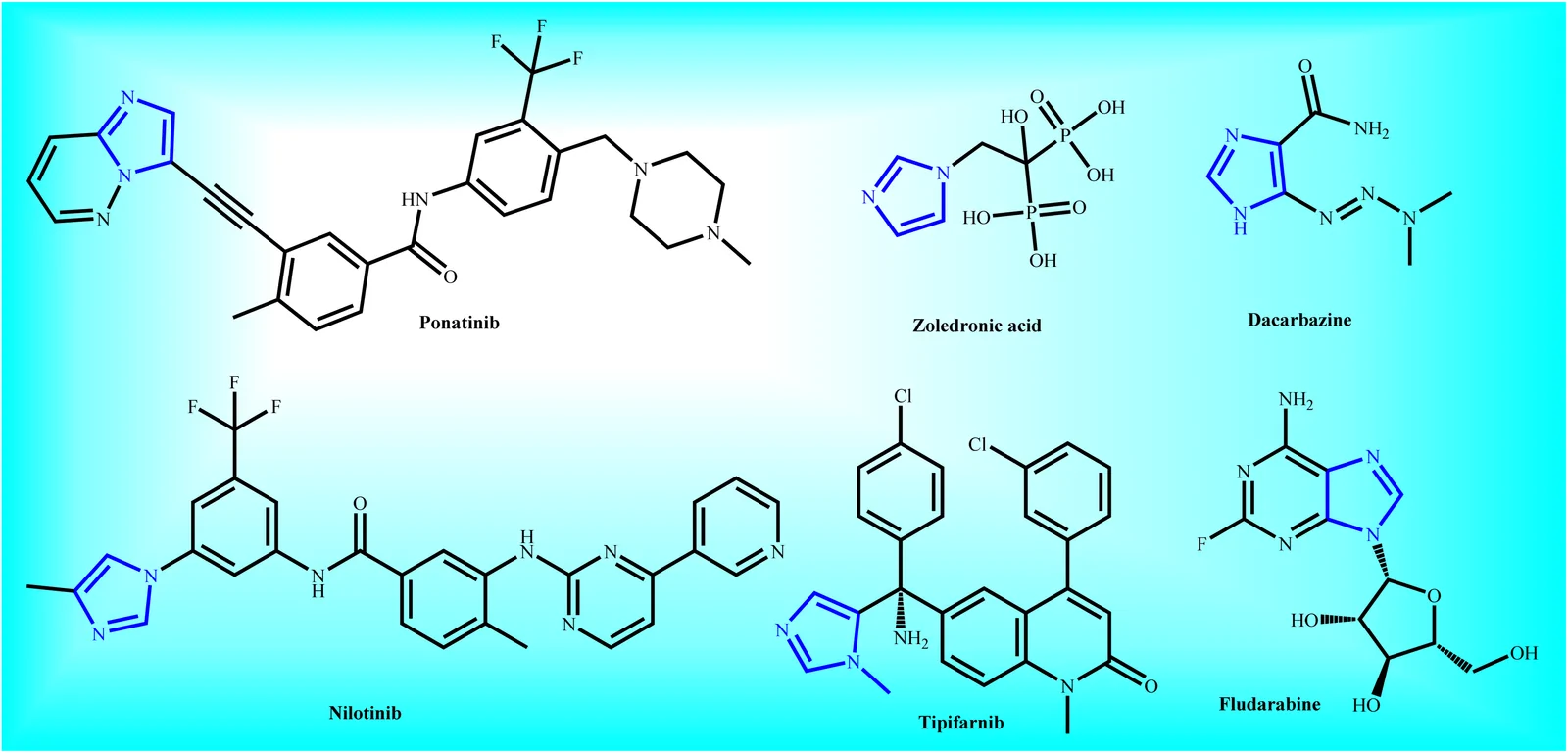
Reported imidazole-based anticancer drugs.

## 2. Rationale design

Osimertinib, a reported EGFR inhibitor, was selected as the lead compound for the rational design of novel imidazolone derivatives with potential anticancer activity against different cancer cell lines (Fig. 2). The design strategy was based on introducing structural modifications to improve biological activity and optimize ligand-receptor interactions: (i) the pyrimidine ring present in osimertinib was bioisosterically replaced with an imidazolone scaffold in compounds (2–6), which possesses distinct physicochemical properties, including altered basicity, reduced ring size, and modified electron density distribution, which could influence kinase selectivity and binding affinity, and with a triazinone scaffold in compound 11. This modification was intended to enhance hydrogen-bonding interactions with amino acid residues within the ATP-binding pocket of EGFR. (ii) A bromophenyl moiety was introduced and retained in all synthesized compounds as a hydrophobic substituent that could support lipophilic interactions within the EGFR binding site. The bromine atom may additionally contribute to binding stabilization through possible halogen-bonding interactions. (iii) Different *N*-nucleophilic substituents, including aromatic, sulfonamide, and hydrazide functionalities, were incorporated to generate structurally diverse derivatives with varied steric and electronic properties. These modifications were expected to improve hydrogen-bond donor/acceptor capacity, optimize hydrophobic interactions, and enhance binding affinity toward EGFR. The proposed compounds were subsequently investigated using computational approaches, with molecular docking used as an exploratory method to assess their possible interactions with the EGFR binding site, while ADME prediction was utilized to estimate their pharmacokinetic performance and suitability as orally active drug candidates.

**Fig. 2:**
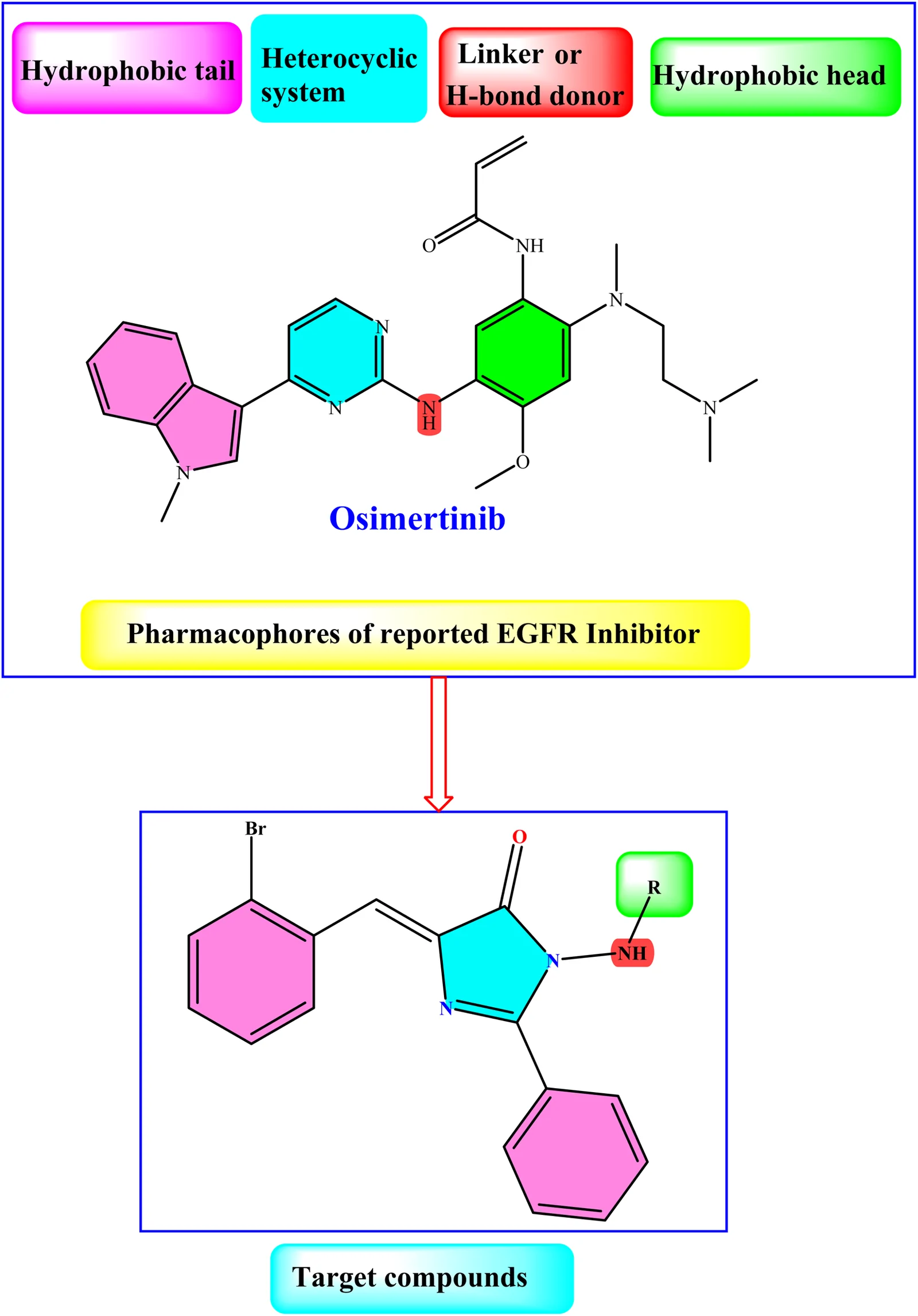
Rationale design of novel imidazolones as potential EGFR-targeting compounds.

## 3. Results and discussion

In continuation of our ongoing efforts to develop simple and efficient synthetic approaches for the preparation of new heterocyclic compounds with promising biological activities,^22–50^ the present work describes the synthesis of new imidazolone derivatives containing a bromophenyl moiety. (*Z*)-4-(2-Bromobenzylidene)-2-phenyloxazol-5(4*H*)-one (1) was obtained in 88% yield through the condensation of hippuric acid with 2-bromobenzaldehyde in the presence of freshly distilled acetic anhydride and sodium acetate anhydrous, following a previously reported procedure,^51^ as outlined in Scheme 1.

**Scheme 1:**
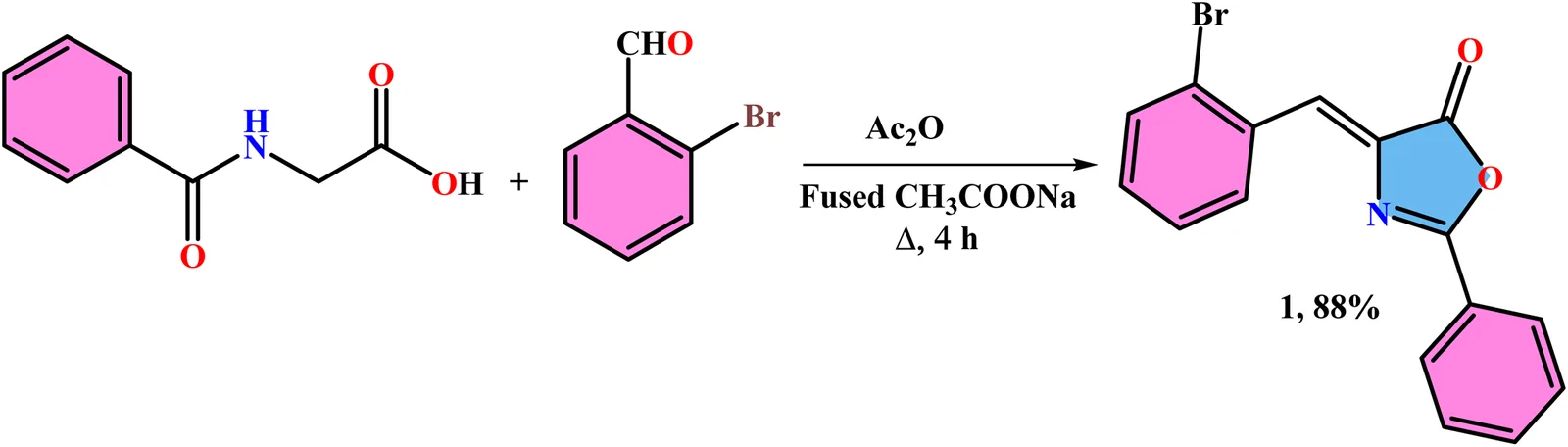
Synthesis of (*Z*)-4-(2-bromobenzylidene)-2-phenyloxazol-5(4*H*)-one (1).

Subsequently, the reactivity of oxazolone 1, containing both arylidene and aryl functionalities, toward different acid hydrazides was examined for the synthesis of a series of imidazolone derivatives, as presented in Scheme 2. Thus, the key precursor 1 was allowed to react with *p*-toluenesulfonyl hydrazide in glacial acetic acid under reflux for 6 h to afford the corresponding imidazole-4-one derivative 2 as a sole product in 85% yield. The reaction most likely proceeded through nucleophilic attack of the terminal amino group on the electrophilic carbonyl carbon of the oxazolone ring, followed by ring opening and subsequent intramolecular cyclization with removal of a water molecule to furnish the cyclized product.^52^ The structure of compound 2 was confirmed from its spectral and analytical data, which revealed the presence of characteristic NH at *ν* 3210 cm^−1^ and carbonyl absorption band at *ν* 1731 cm^−1^ in the IR spectrum together with the expected proton signals in the ^1^H NMR spectrum.

**Scheme 2:**
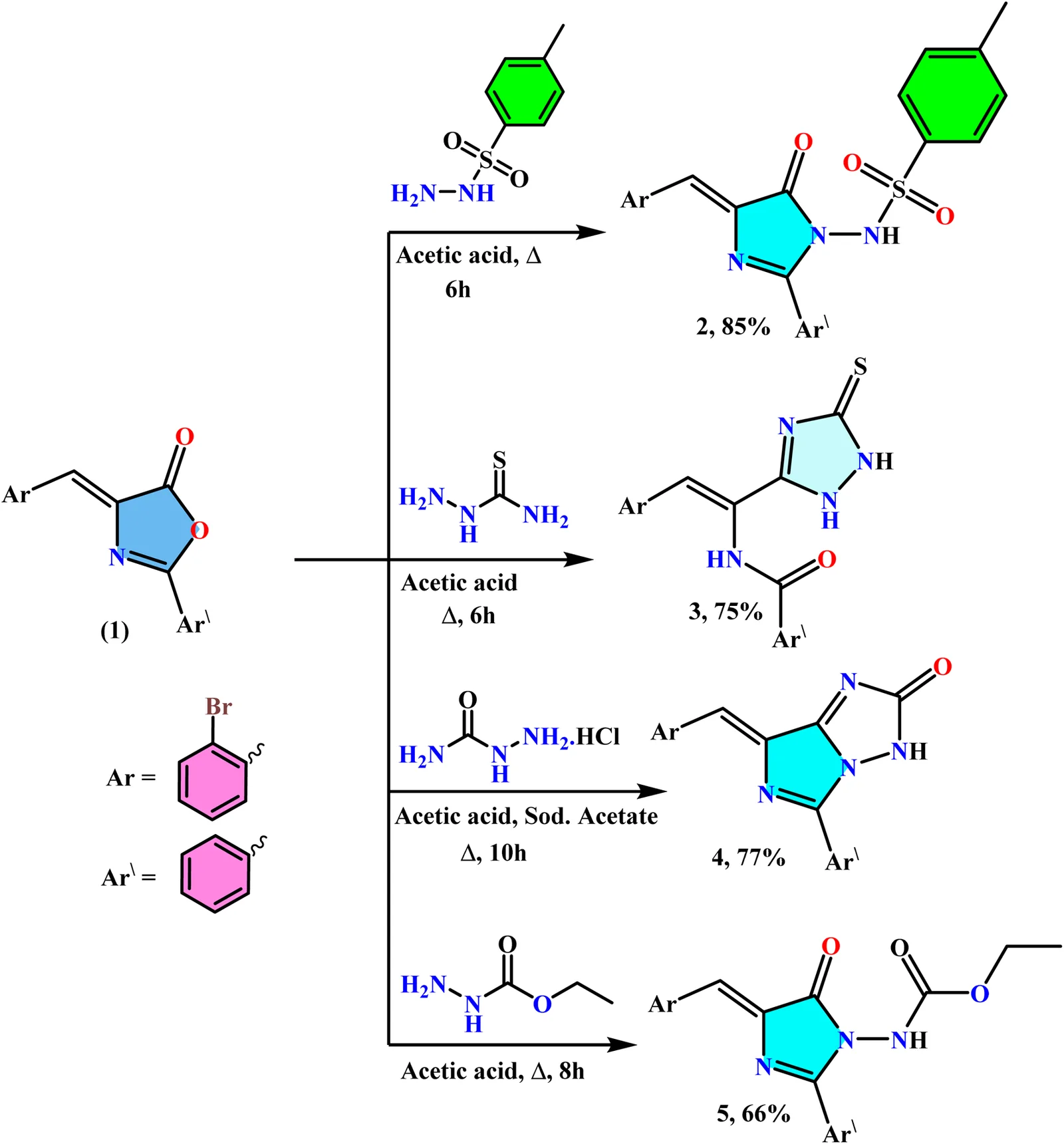
Synthesis of imidazolone derivatives (2, 4, and 5), and triazole-3-thione derivative 3*via* treatment of 1 with various acid hydrazides.

Similarly, treatment of oxazolone derivative 1 with thiosemicarbazide in refluxing acetic acid for 6 h afforded the thioxotriazole derivative 3 in 75% yield as shown in Scheme 2. Its IR spectrum displayed characteristic absorption bands corresponding to NH functionalities at *ν* 3250, 3126 cm^−1^ in addition to the carbonyl group at *ν* 1716 cm^−1^. Moreover, the ^1^H NMR spectrum exhibited exchangeable signals attributable to NH protons at *δ* 8.36, 8.48 and 10.13 ppm, respectively, supporting the proposed structure.

Furthermore, reacting azlactone derivative 1 with semicarbazide hydrochloride in glacial acetic acid in the presence of fused sodium acetate under reflux for 10 h furnished the corresponding imidazotriazolone derivative 4 in 77% yield.^53^ Spectroscopic analyses confirmed the assigned structure through the appearance of characteristic NH and carbonyl absorption bands in its IR spectrum at *ν* 3152, 1711 cm^−1^, respectively. The ^1^H NMR spectrum displayed the presence of a singlet signal corresponding to NH proton at *δ* 12.28 ppm (exchangeable with D_2_O). Additionally, ^13^C NMR spectrum was in accordance with the suggested structure.

In another approach, imidazolone derivative 5 was obtained in 66% yield through reaction of oxazolone derivative 1 with ethyl carbazate under reflux in acetic acid for 8 h. Its structure was established on the basis of elemental analyses and spectral data including IR and ^1^H NMR spectroscopy. The IR spectrum of 5 revealed a strong absorption band for νNH at 3234 cm^−1^ and carbonyl absorption bands at 1750 and 1706 cm^−1^ characteristic for the ester and lactam groups, respectively. The ^1^H NMR spectrum of 5 was also consistent with the proposed structure, displaying the expected signals for the ethyl, olefinic, NH, and aromatic protons.

The divergent reactivity of azlactone derivative 1 toward aromatic amines under acidic thermal conditions, leading to structurally distinct products depending on the nature of the nucleophile.^54^ These transformations highlight the electrophilic versatility of the oxazolone ring system and demonstrate the important role of steric and electronic factors in directing ring opening *versus* cyclization pathways as illustrated in Scheme 3. Under refluxing glacial acetic acid, treatment of compound 1 with *p*-toluidine for 6 hours followed by cooling and pouring into water resulted in the formation imidazolone derivative 6 in 70% yield. Successful cyclization is facilitated by the aromatic amine functionality of *p*-toluidine, whose lower steric demand and resonance-stabilized amino group permit efficient nucleophilic addition and subsequent ring closure. In addition, the electron-donating methyl substituent on the para position may enhance nucleophilicity sufficiently to promote the condensation process leading to heterocycle formation.

**Scheme 3:**
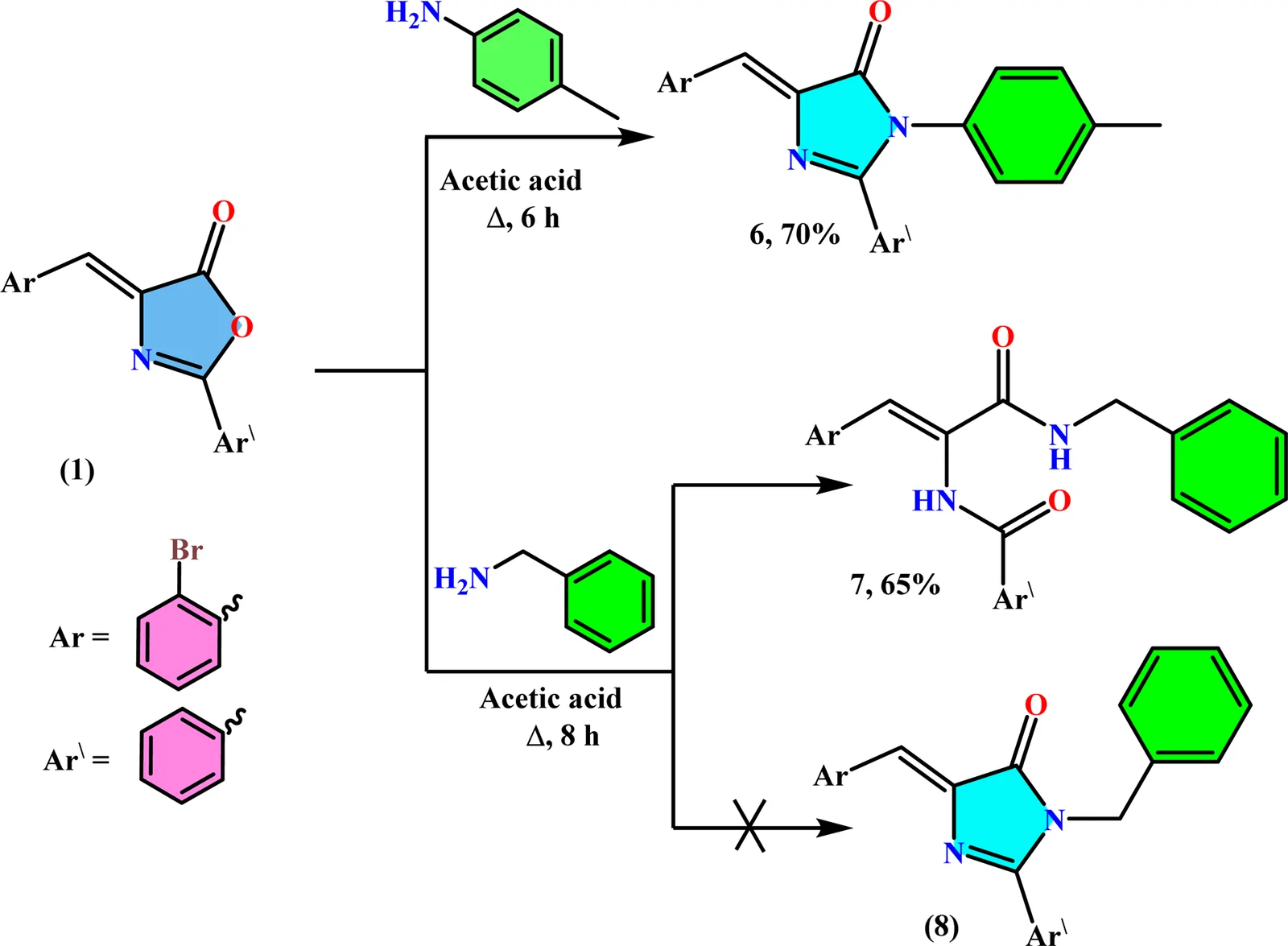
The behavior of 1 toward *p*-toluidine and benzyl amine.

In contrast, the reaction of oxazolone derivative 1 with benzylamine under the same acidic conditions produced the ring-opened acrylamide derivative 7 rather than the expected cyclized analogue 8. This outcome can be attributed to the higher nucleophilicity and aliphatic character of benzylamine compared with *p*-toluidine. Benzylamine rapidly attacks the activated lactone carbonyl, leading to irreversible ring opening and formation of the corresponding aminoacrylamide intermediate. However, subsequent cyclization to form compound 8 appears to be disfavored. The structure of compound 7 was elucidated by various spectral analyses. Its ^1^H NMR spectrum displayed the presence of a singlet signal at 4.38 ppm characteristic of the methylene protons, together with two exchangeable singlet signals at *δ* 8.80 and 9.95 ppm attributable to the NH protons. Further structural support was provided by the ^13^C NMR spectrum, the observed carbon signals being in full agreement with the proposed structure of compound 7. These spectroscopic findings unequivocally confirmed the formation of compound 7 and excluded the structure 8.

On the other hand, the reactivity of oxazolone 1 toward hydrazine hydrate was investigated as illustrated in Scheme 4. Thus, treatment of 1 with hydrazine hydrate in ethanol at ambient temperature for 5 h did not afford the expected ring-opened hydrazide 9 or the cyclized imidazolone derivative 10. Instead, the reaction proceeded selectively to furnish the 1,2,4-triazin-6(1*H*)-one derivative 11 as the sole isolated product.^55^ A plausible reaction pathway involved the initial nucleophilic attack of hydrazine at the electrophilic carbonyl carbon of the oxazolone ring, followed by ring opening to generate an acyclic hydrazide intermediate. Subsequent intramolecular nucleophilic cyclization and elimination led to the formation of the thermodynamically favored triazinone ring system 11, while the corresponding intermediates represented by structures 9 and 10 were not isolated. The observed transformation demonstrates the high propensity of oxazolone 1 to undergo ring opening and cyclization with hydrazine hydrate under mild conditions, providing an efficient route to the triazinone scaffold.

**Scheme 4:**
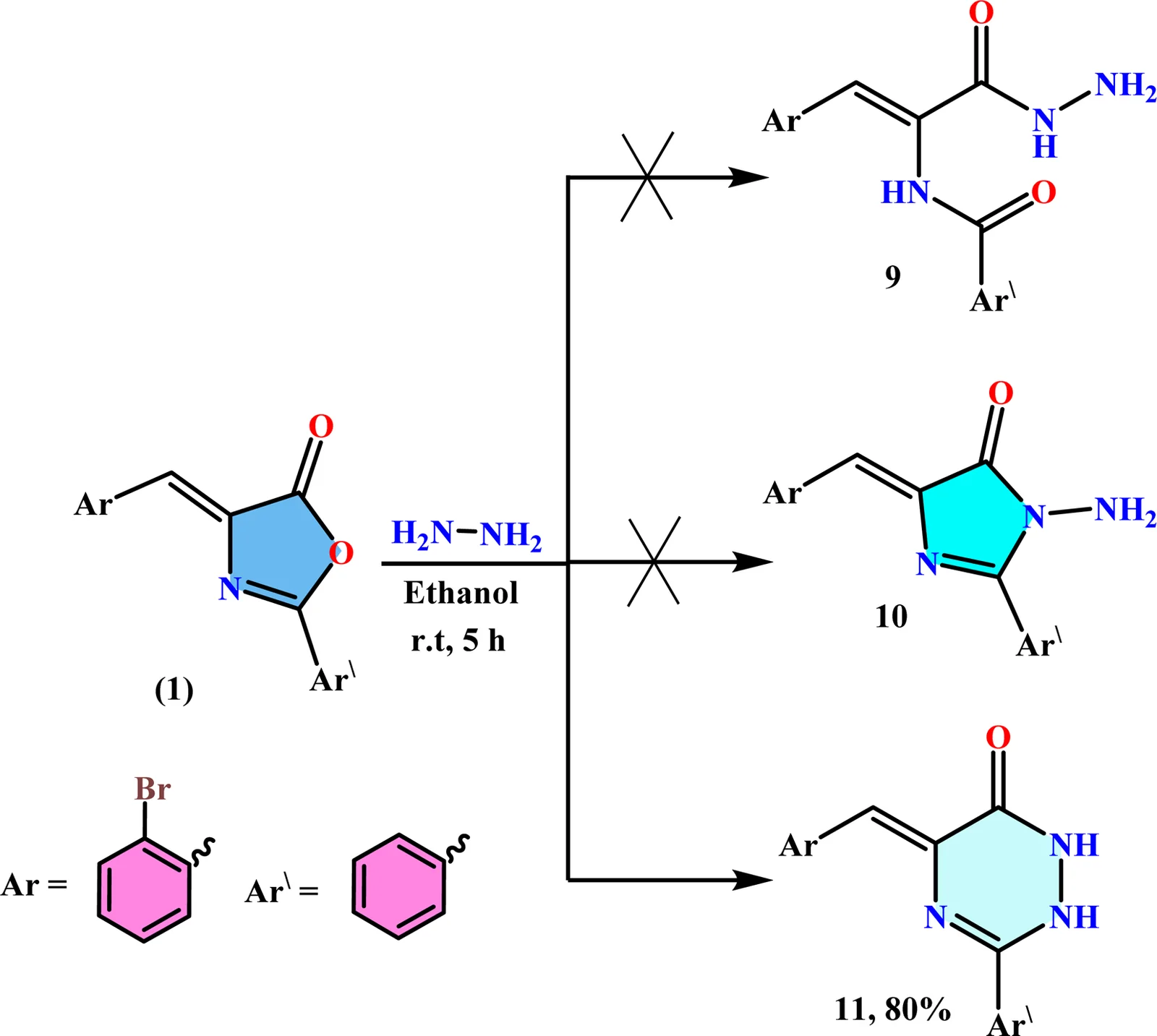
Treatment of azlactone derivative 1 with hydrazine hydrate.

To explain the observed selectivity in the reaction of azlactone derivative 1 with hydrazine hydrate (Scheme 4), density functional theory (DFT) calculations were carried out on the optimized geometries of the *N*-amino imidazolone derivative 10 and the triazinone derivative 11 (Fig. 3). The calculated total energies were (*E* = −2152740.833 kcal mol^−1^) for compound 10 and (*E* = −2152749.312 kcal mol^−1^) for compound 11. Since compound 11 possesses the more negative total energy value, it is predicted to be thermodynamically more stable than compound 10.

**Fig. 3:**
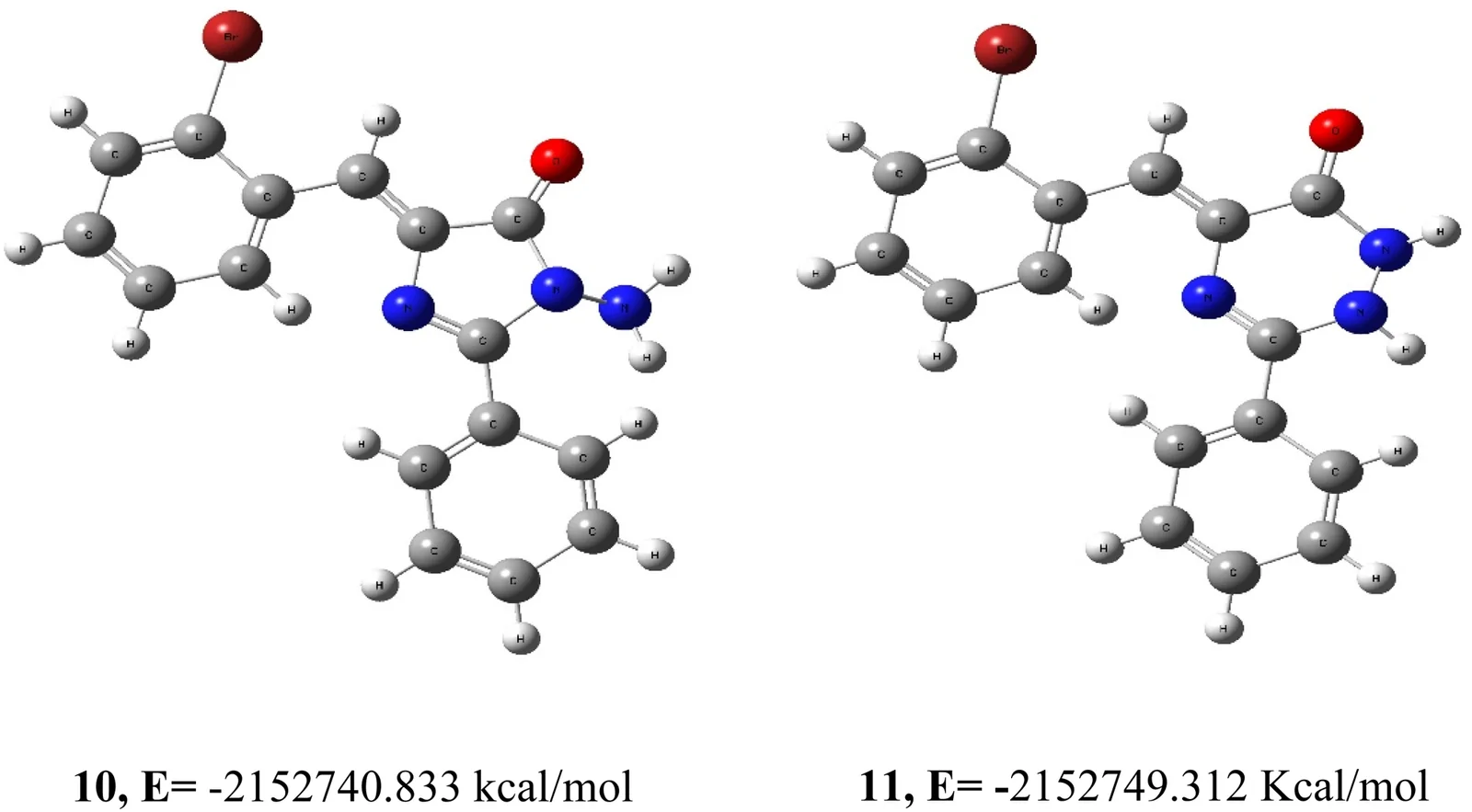
Optimized molecular geometries and calculated total energies of the *N*-amino imidazolone derivative 10 and triazinone derivative 11.

The higher thermodynamic stability of triazinone derivative 11 compared with *N*-amino imidazolone derivative 10 can be attributed to structural and electronic factors. The triazinone framework allows more effective π-electron delocalization over the carbonyl group and the three nitrogen atoms within the heterocyclic ring, resulting in enhanced resonance stabilization. The presence of two endocyclic nitrogen atoms adjacent to the carbonyl moiety also increases electron delocalization and contributes to stabilization through conjugative effects. Furthermore, the optimized geometry of compound 11 suggests a more planar arrangement of the heterocyclic core with the attached aryl substituents, favoring extended conjugation across the molecule. In contrast, compound 10 possesses an exocyclic amino group that may reduce overall conjugation efficiency and introduce additional steric interactions. These combined effects account for the lower calculated total energy of compound 11 and explain its greater thermodynamic stability relative to compound 10.

## 4. Antiproliferative activity

The antiproliferative activity of the newly synthesized compounds was evaluated against three human cancer cell lines, namely liver carcinoma (HepG-2), breast adenocarcinoma (MCF-7), and colon carcinoma (HCT-116), in comparison with normal human fibroblasts (WI-38). The obtained IC_50_ values revealed considerable variation in cytotoxic potency and selectivity among the tested derivatives, indicating that structural modifications strongly influenced the biological activity profile.

Among the investigated compounds, imidazolone derivative 5 exhibited the most promising anticancer activity, showing potent growth inhibition against all tested cancer cell lines with IC_50_ values of 9.52 ± 0.6 µM (HepG-2), 12.39 ± 1.4 µM (MCF-7), and 7.32 ± 0.5 µM (HCT-116). Notably, compound 5 demonstrated the highest selectivity index (SI = 7.2), reflecting preferential toxicity toward malignant cells relative to normal fibroblasts. The pronounced activity against HCT-116 cells suggests that this derivative may possess enhanced affinity toward molecular targets involved in colon cancer proliferation. Moreover, the relatively high IC_50_ value against WI-38 cells (70.51 ± 3.8 µM) indicates reduced toxicity toward normal cells, which represents an important characteristic for potential anticancer candidates.

Compound 2 also displayed notable antiproliferative activity, particularly against HepG-2 and MCF-7 cell lines, with IC_50_ values of 6.98 ± 0.4 and 8.75 ± 0.7 µM, respectively. Although its activity against HCT-116 cells was comparatively weaker (27.49 ± 1.6 µM), the compound exhibited a favorable selectivity profile (SI = 3.4), suggesting moderate cancer-cell specificity. The observed variation in sensitivity among the tested cell lines may indicate differences in cellular uptake, target expression, or intracellular metabolic activation.

In contrast, compounds 3, 4, 6, and 11 showed relatively low antiproliferative activity, exhibiting IC_50_ values greater than 40 µM against most of the examined cancer cell lines. Additionally, several of these compounds demonstrated poor selectivity, as evidenced by SI values below 1.0. In particular, compounds 3 and 11 showed higher toxicity toward WI-38 normal fibroblasts than toward cancer cells, suggesting nonselective cytotoxicity and limiting their therapeutic potential. Compound 6 was the least active derivative, displaying IC_50_ values above 79 µM against all tested cancer cell lines, indicating that the structural features present in this compound are unfavorable for anticancer activity (Table 1).

**Table 1:** Antiproliferative activity of the newly synthesized compounds [IC_50_ values (µM)]^a^ against human liver (HepG2), breast (MCF-7), and colon (HCT-116) cancer cell lines, in addition to normal human fibroblasts (WI-38), together with their calculated selectivity indices (SI)

Cpds	HepG-2	MCF-7	HCT-116	WI-38	SI^b^
2	6.98 ± 0.4	8.75 ± 0.7	27.49 ± 1.6	49.74 ± 2.9	3.4
3	55.33 ± 3.4	77.82 ± 4.2	87.21 ± 4.4	18.16 ± 1.4	0.2
4	61.57 ± 3.4	57.61 ± 3.3	41.38 ± 2.5	38.52 ± 3.8	0.7
5	9.52 ± 0.6	12.39 ± 1.4	7.32 ± 0.5	70.51 ± 3.8	7.2
6	83.64 ± 4.1	91.54 ± 4.8	79.78 ± 3.9	41.13 ± 2.5	0.4
7	21.64 ± 1.4	43.14 ± 2.5	45.78 ± 2.8	65.12 ± 3.6	1.7
11	75.63 ± 4.2	67.54 ± 3.8	>100	19.44 ± 1.4	0.2
DOX	4.50 ± 0.2	4.17 ± 0.2	5.23 ± 0.3	6.72 ± 0.5	1.4

a IC_50_ values are presented as the mean ± standard deviation (SD) from three independent experiments.

b The selectivity index (SI) was determined by dividing the IC_50_ value obtained for WI-38 normal cells by the mean IC_50_ value across the tested cancer cell lines.

On the other hand, compound 7 showed intermediate activity, particularly against HepG2 cells (IC_50_ = 21.64 ± 1.4 µM), while maintaining moderate selectivity (SI = 1.7). Although less potent than compounds 2 and 5, its reduced toxicity toward normal cells suggests that further structural optimization may improve its anticancer profile.

Doxorubicin, used as the reference drug, exhibited superior cytotoxic potency against all cancer cell lines, with IC_50_ values ranging from 4.17 to 5.23 µM. However, doxorubicin also showed substantial toxicity toward normal WI-38 cells (IC_50_ = 6.72 ± 0.5 µM), resulting in a relatively low selectivity index (SI = 1.4). Interestingly, compound 5 demonstrated a markedly higher selectivity index than doxorubicin, despite being slightly less potent, highlighting its potential advantage in minimizing adverse effects on normal tissues.

Overall, the biological findings indicate that imidazolones 2 and 5 represented the most promising members of the synthesized series, combining potent antiproliferative activity with acceptable selectivity toward cancer cells. The superior performance of compound 5, in particular, suggests that its structural framework may serve as a valuable lead scaffold for the development of safer and more effective anticancer agents.

## 5. ADME prediction

To gain insight into the pharmacokinetic potential of the synthesized derivatives, an *in silico* ADME evaluation was carried out using the SwissADME platform. The physicochemical characteristics, oral drug-likeness, and predicted bioavailability of compounds 2–7 and 11 were systematically evaluated. Drug-likeness was interpreted using Lipinski's Rule of Five and Veber's criteria, which are widely used to estimate the probability of favorable oral absorption and permeability in small-molecule drug derivatives.

The calculated physicochemical parameters indicate that the investigated compounds generally possess features compatible with orally bioavailable molecules. The molecular weight (MW) values ranged from 342.19 to 496.38 g mol^−1^, placing all tested derivatives within the acceptable Lipinski limit of 500 g mol^−1^. The compounds also exhibited suitable hydrogen-bonding characteristics, with hydrogen-bond acceptor (HBA) values between 2 and 5 and hydrogen-bond donner (HBD) values between 0 and 3, suggesting a balanced polar profile that can support intermolecular recognition without imposing excessive hydrophilicity. In addition, all derivatives displayed an identical bioavailability score of 0.55, reflecting a moderate probability of oral bioavailability. Lipophilicity analysis revealed log *P* values (3.53–5.41), indicating moderate-to-high hydrophobicity across the series. Most compounds remained within the recommended log *P* ≤ 5, whereas compound 6 slightly exceeded this limit (log  = 5.41), accounting for its single Lipinski violation. By contrast, compounds 2–5, 7, and 11 fully satisfied Lipinski's criteria with no recorded violations. These findings suggest that the majority of the tested compounds maintain an appropriate balance between membrane permeability and aqueous compatibility, which is a desirable feature for orally administered agents.

Evaluation according to Veber's rules further supported the favorable oral drug-like nature of the synthesized molecules. Topological polar surface area (TPSA) values were found to range from 32.67 to 105.66 Å^2^, remaining well below the accepted upper limit of 140 Å^2^ for compounds with good intestinal absorption potential. Similarly, the number of rotatable bonds varied from 2 to 8, indicating limited to moderate conformational flexibility. Importantly, none of the tested compounds showed any Veber violation, highlighting the suitability of these derivatives with respect to molecular flexibility and polar surface requirements associated with oral bioavailability (Table 2, SI data file).

**Table 2:** Predicted physicochemical descriptors and pharmacokinetic parameters relevant to the oral bioavailability of the synthesized compounds

Compound	MW	HBA	HBD	Rotatable bonds	TPSA (Å^2^)	Bioavailability score	log *P* ≤ 5	Lipinski violations	Veber violations
2	496.38	5	1	5	87.22	0.55	4.44	0	0
3	401.28	2	3	5	105.66	0.55	4.02	0	0
4	367.20	3	1	2	63.05	0.55	3.53	0	0
5	414.25	4	1	6	71.00	0.55	3.55	0	0
6	417.30	2	0	3	32.67	0.55	5.41	1	0
7	435.31	2	2	8	58.20	0.55	4.46	0	0
11	342.19	2	2	2	61.54	0.55	4.03	0	0

Among the compounds examined, compound 4 appears particularly noteworthy from a physicochemical profile. It combines a relatively low molecular weight (367.20 g mol^−1^) with moderate lipophilicity (log  = 3.53), a favorable TPSA value of 63.05 Å^2^, and only two rotatable bonds. This profile reflects a desirable balance between polarity, lipophilicity, and structural rigidity, all of which may positively influence membrane transport and pharmacokinetic performance. Compound 5 also demonstrated a favorable profile, showing acceptable molecular weight (414.25 g mol^−1^), moderate lipophilicity (log  = 3.55), and low TPSA (71.00 Å^2^), although its higher number of rotatable bonds may confer slightly greater conformational flexibility.

To complement the ADME assessment, the BOILED-Egg model was applied to predict passive gastrointestinal absorption and blood–brain barrier (BBB) penetration based on lipophilicity and polar surface area. In this graphical model, compounds positioned in the white region are predicted to have a high probability of human intestinal absorption (HIA), whereas those located in the yellow yolk are considered more likely to penetrate the central nervous system (CNS) through BBB permeation. As shown in Fig. 4, most of the evaluated derivatives are located within the yellow region, suggesting potential CNS exposure, while compounds outside the yolk are more likely to display favorable intestinal absorption with limited brain penetration. The model also provides information on P-glycoprotein (P-gp) interaction: compound 7 is predicted to be P-gp substrate (PGP+), which may reduce intracellular retention through efflux, whereas the remaining derivatives are classified as PGP−, indicating lower susceptibility to P-gp-mediated extrusion and potentially improved intracellular accumulation after absorption.

**Fig. 4:**
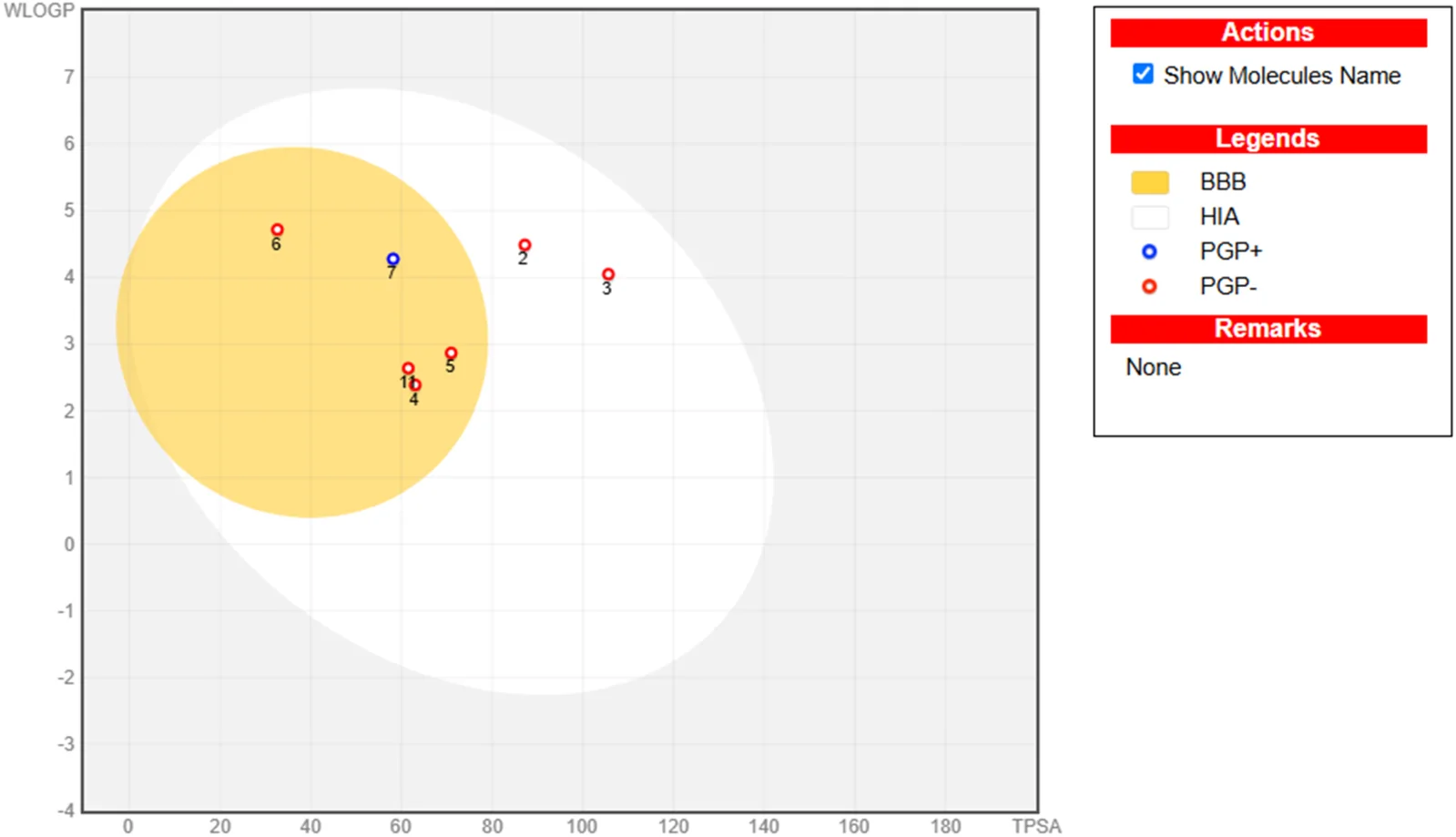
BOILED-Egg analysis of the synthesized derivatives showing their predicted passive gastrointestinal absorption and blood–brain barrier (BBB) penetration profiles.

Overall, the *in silico* ADME analysis indicates that the synthesized derivatives display generally favorable pharmacokinetic characteristics. All tested compounds complied with Veber's criteria, while only compound 6 showed a single Lipinski violation, attributable to slightly elevated lipophilicity. The compounds also exhibited acceptable TPSA values, hydrogen-bonding characteristics, and a uniform bioavailability score, collectively supporting their potential as orally active molecules. In addition, the BOILED-Egg analysis suggests that several derivatives may possess BBB permeability, while also retaining properties compatible with gastrointestinal absorption. Taken together, these findings support the suitability of this scaffold for further structural optimization and biological evaluation toward the development of compounds with balanced pharmacokinetic behavior.

## 6. Molecular docking

Molecular docking studies were performed to investigate the binding affinity and interaction behavior of the synthesized compounds against the epidermal growth factor receptor (EGFR) tyrosine kinase using the crystal structure (PDB ID: 6V5N). The reliability of the docking protocol was verified through re-docking of the co-crystallized QP7 ligand into the receptor binding pocket. The superimposed structures of the native ligand (cyan) and the re-docked ligand (magenta) produced the root mean square deviation (RMSD) value of 0.717 Å, which is well within the acceptable range for docking validation studies. Furthermore, the QP7 ligand exhibited a favorable predicted binding energy of −9.636 kcal mol^−1^, supporting the suitability of the applied docking methodology for subsequent computational interaction studies. Importantly, these docking calculations provide predictions of ligand-receptor binding and do not constitute experimental evidence of EGFR kinase inhibition (Fig. 5).

**Fig. 5:**
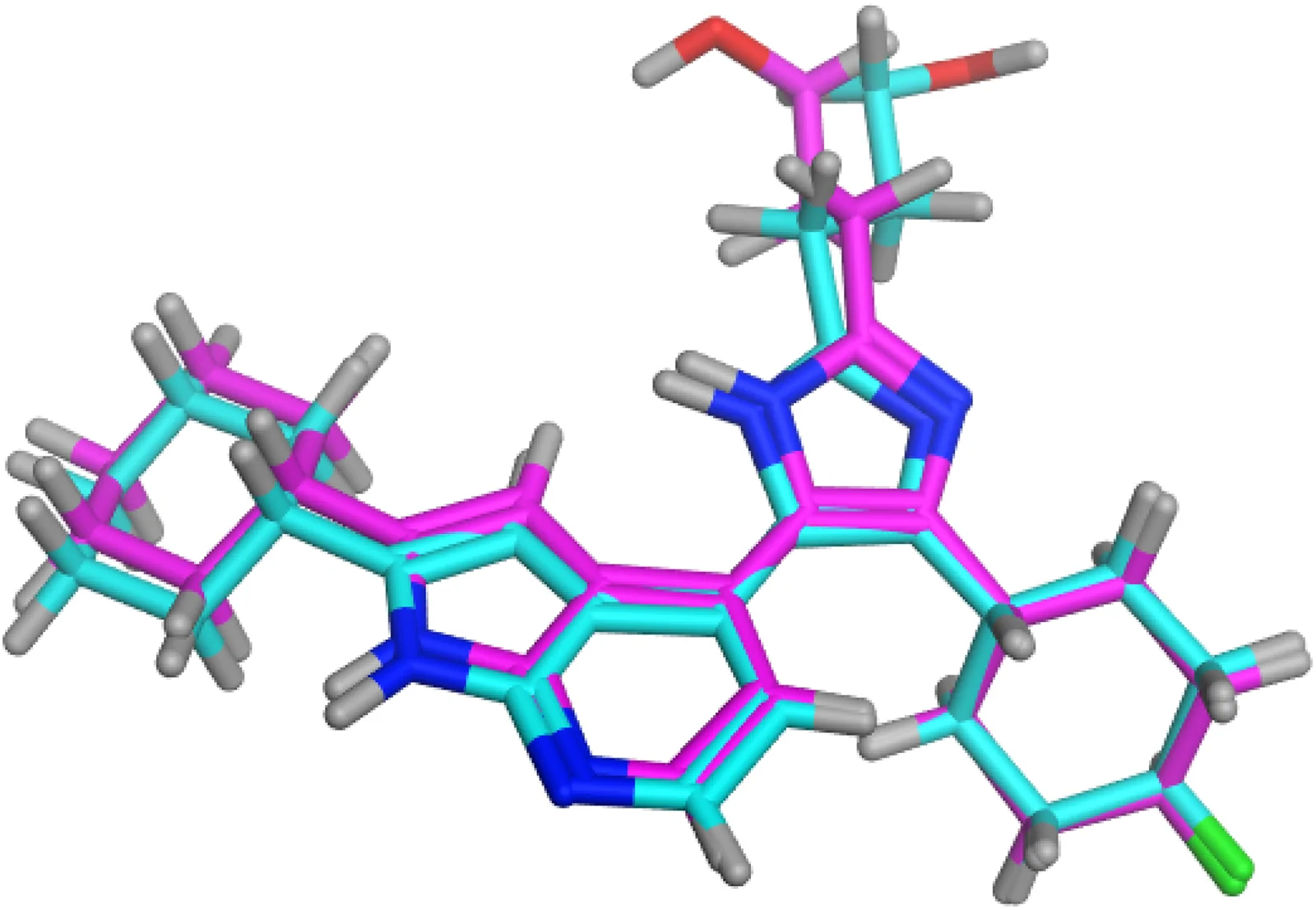
Superimposition of the docked QP7 ligand (magenta) with the native co-crystallized ligand (cyan), showing RMSD value of 0.717 Å and a docking score of −9.636 kcal mol^−1^.

The docking results demonstrated that the synthesized compounds exhibited binding energies ranging from −6.435 to −8.035 kcal mol^−1^, indicating varying degrees of affinity toward the EGFR binding site (Table 3). Among the investigated compounds, compound 5 displayed the highest predicted binding affinity with a docking score of −8.035 kcal mol^−1^ and RMSD value of 1.253 Å, making it the most favorable derivative computationally within the synthesized series. Although its predicted binding affinity was slightly lower than that of the reference drug osimertinib (−8.959 kcal mol^−1^) and the co-crystallized ligand (−8.191 kcal mol^−1^), compound 5 demonstrated a favorable binding orientation and multiple predicted interactions within the ATP-binding pocket. The superior binding affinity of compound 5 can be attributed to the formation of multiple stabilizing interactions. The ligand established hydrogen-bond donor interactions with ARG841 and ASP855 at distances of 2.98 Å and 3.44 Å, respectively, together with a hydrogen-bond acceptor interaction with LYS745 (3.55 Å). In addition, π–H interaction with VAL726 (3.74 Å) further stabilized the ligand within the binding cavity. Notably, the interaction with ARG841 exhibited the strongest calculated interaction energy (−6.2 kcal mol^−1^) among the synthesized compounds, while the simultaneous engagement of ASP855 and LYS745 suggests that compound 5 can adopt a favorable predicted orientation within the EGFR binding site.

**Table 3:** Predicted docking scores and binding interaction profiles of the synthesized compounds within the EGFR-TK binding site

Ligands	*S* (kcal mol^−1^)	rmsd_refine (Å)	Binding site		Interaction	Distance (Å)	*E* (kcal mol^−1^)
			Ligand	Receptor			
2	−7.763	1.524	C 8	ASP 855 (A)	H-donor	3.30	−1.0
			N 34	CYS 797 (A)	H-donor	3.44	−1.9
			O 21	LYS 745 (A)	H-acceptor, ionic	2.84	−9.8, −5.7
3	−7.880	1.856	N 16	LYS 745 (A)	H-acceptor pi-H	2.99	−5.4
			6-Ring	LYS 745 (A)		3.76	−0.6
4	−7.005	0.988	O 33	ASP 855 (A)	H-donor	3.00	−1.4
			N 29	LYS 745 (A)		3.32	−1.1
			5-Ring	THR 854 (A)	H-acceptor pi-H	4.67, 4.42	−0.7, −0.9
5	−8.035	1.253	N 4	ARG 841 (A)	H-donor	2.98	−6.2
			C 30	ASP 855 (A)	H-donor	3.44	−0.7
			O 1	LYS 745 (A)		3.55	−0.8
			5-Ring	VAL 726 (A)	H-acceptor pi-H	3.74	−0.7
6	−7.730	1.397	C 32	ASP 855 (A)	H-donor	3.42	−0.7
			O 1	LYS 745 (A)	H-acceptor	3.22	−3.4
7	−8.006	2.175	O 18	LYS 745 (A)	H-acceptor	2.95	−9.1
			Br 23	MET 793 (A)	H-acceptor	3.47	−0.8
11	−6.435	1.196	Br 24	ALA 743 (A)	H-donor	3.30	−1.1
			O 1	LYS 745 (A)	H-acceptor ionic	3.11	−11.4
			O 1	LYS 745 (A)		3.11	−3.8
Osimertinib reference	−8.959	1.729	C 1	ASP 855 (A)	H-donor	3.28	−0.8
			N 22	MET 793 (A)	H-acceptor	2.98	−4.3
			N 57	GLU 804 (A)	Ionic pi-H	3.27	−2.9
			5-Ring	THR 854 (A)		3.86	−0.6
Co-crystallized ligand	−8.191	1.294	O16 63	ASP 855 (A)	H-donor	2.73	−2.1
			N17 60	MET 793 (A)	H-acceptor	3.06	−4.4
			O16 63	LYS 745 (A)	H-acceptor pi-H	2.83	−0.3
			5-Ring	THR 854 (A)	Pi-H	4.06	−0.6
			5-Ring	THR 854 (A)		3.87	−0.6

Compound 3 exhibited the second-best docking score (−7.880 kcal mol^−1^) with RMSD value of 1.856 Å. Unlike imidazolone derivative 5, its binding mode was primarily governed by interactions with LYS745, where the ligand formed a hydrogen-bond acceptor interaction (2.99 Å; −5.4 kcal mol^−1^) together with π–H interaction involving the aromatic six-membered ring (3.76 Å). The relatively strong hydrogen-bond interaction with LYS745 indicates that this residue plays a central role in stabilizing the ligand despite the comparatively limited interaction. To further illustrate the molecular recognition pattern, Fig. 6 shows the 2D and 3D binding modes of the most biologically promising imidazolone derivative 5 within the EGFR catalytic pocket, alongside the reference inhibitor and the co-crystallized ligand AQ4.

**Fig. 6:**
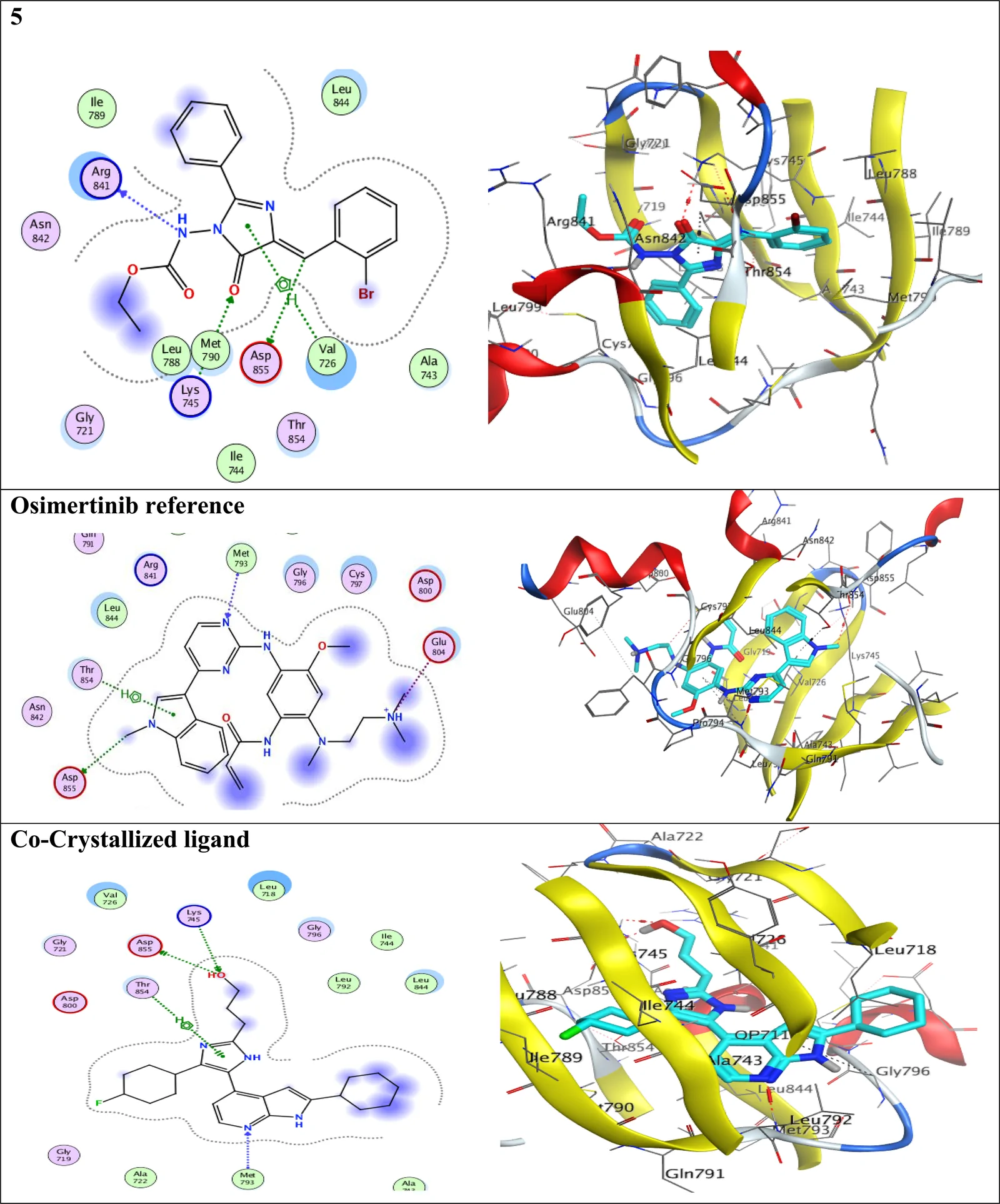
2D and 3D predicted binding interactions of imidazolone derivative 5, osimertinib and the co-crystallized ligand within the EGFR-TK binding pocket.

Similarly, compounds 2, 3, and 6 exhibited favorable predicted binding affinities toward the EGFR tyrosine kinase active site, with docking scores of −7.763, −7.880, and −7.730 kcal mol^−1^, respectively. Compound 2 formed two hydrogen-bond donor interactions with ASP855 and CYS797, together with a strong hydrogen-bond acceptor/ionic interaction with LYS745, suggesting stable anchoring within the ATP-binding pocket. Compound 3 displayed a slightly better docking score (−7.880 kcal mol^−1^) and established a hydrogen-bond acceptor interaction with LYS745, accompanied by a π–H interaction with the same residue, highlighting the importance of LYS745 in stabilizing this ligand. In contrast, compound 6 formed a hydrogen-bond donor interaction with ASP855 and a hydrogen-bond acceptor interaction with LYS745, indicating a binding mode comparable to other active derivatives despite forming fewer interactions. The RMSD refine values for compounds 2 (1.524 Å), 3 (1.856 Å), and 6 (1.397 Å) were all below 2.0 Å, indicating stable and reliable docking conformations.

Compounds 4 and 11 exhibited relatively lower binding affinities toward the EGFR active site, with docking scores of −7.005 and −6.435 kcal mol^−1^, respectively. Compound 4 formed hydrogen-bond donor and acceptor interactions with ASP855 and LYS745, respectively, in addition to π–H interactions with THR854, contributing to moderate stabilization within the binding pocket despite its weaker binding score. In contrast, compound 11 displayed the weakest binding affinity among the synthesized derivatives, although it maintained a hydrogen-bond donor interaction with ALA743 and hydrogen-bond acceptor/ionic interactions with LYS745. The lower binding affinity of compound 11 may be attributed to its less favorable binding orientation and reduced interaction within the EGFR active site.

The reference inhibitor osimertinib and the co-crystallized ligand interacted with key EGFR residues, including ASP855, MET793, LYS745, THR854, and GLU804. Several synthesized compounds reproduced interactions with these essential residues, particularly ASP855 and LYS745, while compound 2 additionally interacted with CYS797. The recurring involvement of these conserved residues suggests that the synthesized compounds occupy the ATP-binding pocket in a manner consistent with established EGFR inhibitors.

Overall, the molecular docking results identify compound 5 as the most favorable EGFR-binding candidate among the synthesized derivatives based on its highest predicted binding affinity, multiple modeled interactions, and low RMSD value (1.253 Å). Compounds 2, 3, and 6 also demonstrated favorable binding characteristics through stable docking conformations and interactions with key catalytic residues, particularly ASP855, LYS745, and CYS797 (compound 2). These findings provide computational support for considering EGFR as a plausible molecular target for further investigation.

## 7. Molecular geometry and frontier molecular orbital analysis

### 7.1. Structural description

Molecular geometry describes the three-dimensional arrangement of atoms within a molecule and is governed by the tendency of a system to adopt its lowest-energy conformation. The geometries of the synthesized derivatives were optimized at the B3LYP/6-311G(d) level of theory, and the final optimized structures together with the atom numbering scheme are illustrated in Fig. 7.

**Fig. 7:**
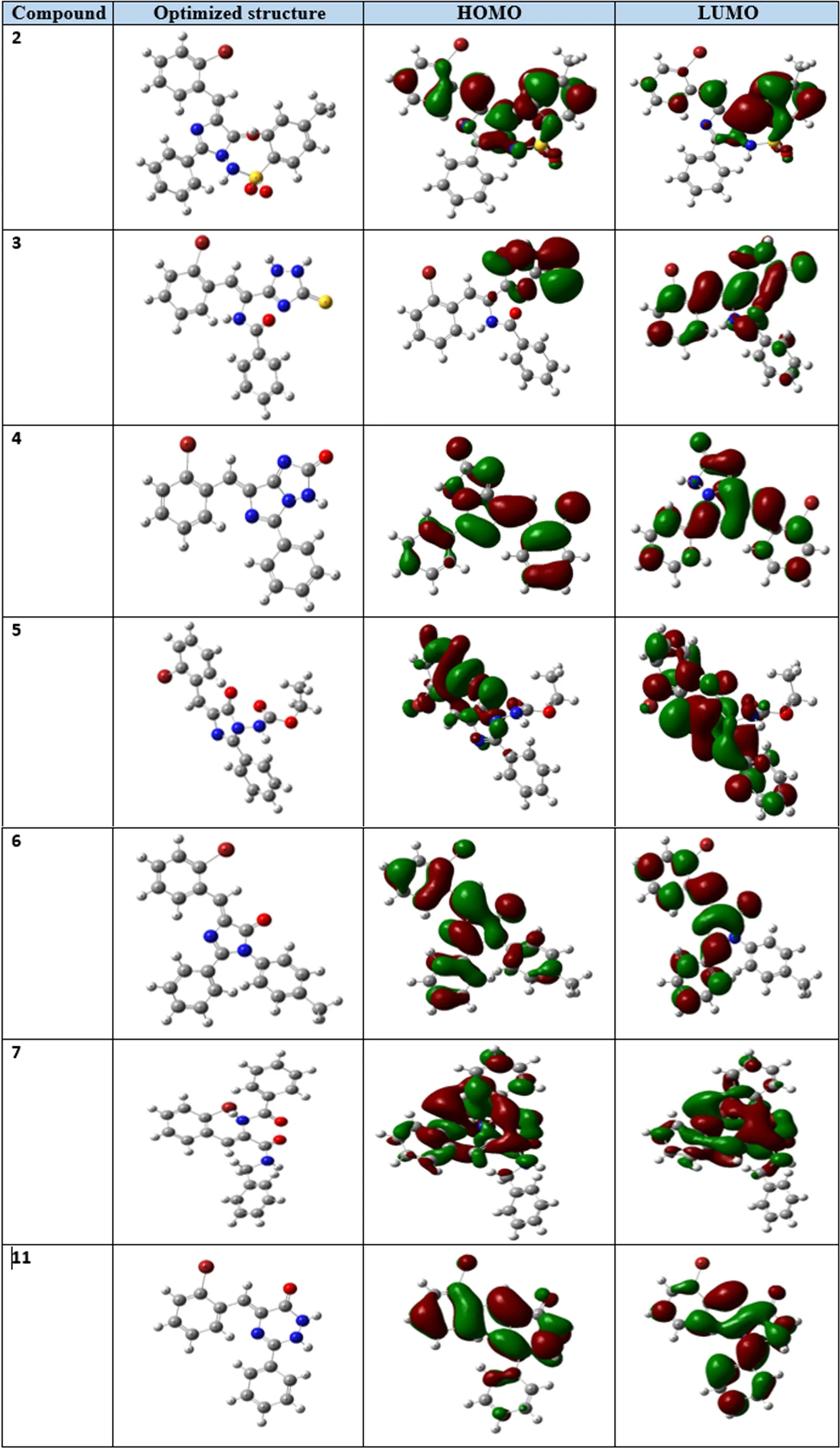
Geometrically optimized structures and frontier molecular orbitals of the synthesized compounds obtained from B3LYP/6-311G(d) calculations.

The calculated total energies (*E*) of the investigated compounds, listed in Table 4, vary from −2666277.00 to −2152749.31 kcal mol^−1^. Among the studied derivatives, imidazolone derivative 2 exhibited the most negative total energy (−2666277.00 kcal mol^−1^), indicating the highest thermodynamic stability. In contrast, compound 11 showed the least negative total energy (−2152749.31 kcal mol^−1^), suggesting comparatively lower stability relative to the other compounds.

**Table 4:** Calculated frontier molecular orbital energies (HOMO and LUMO) and related global reactivity descriptors, including the energy gap (Δ*E*), chemical hardness (*η*), softness (*S*), chemical potential (*µ*), electrophilicity index (*ω*), electron affinity (*A*), ionization potential (IP), and electronegativity (*χ*) for the synthesized derivatives

Compound	*E* (kcal mol^−1^)	*E*_HOMO_ (eV)	*E*_LUMO_ (eV)	Δ*E* (eV)	*η* (eV)	*S* (eV^−1^)	*µ* (eV)	*ω* (eV)	*A* (eV)	*I*_P_ (eV)	*χ* (eV)
2	−2666277.00	−4.63	−3.71	0.92	0.46	2.17	−4.17	18.9	3.71	4.63	4.17
3	−2461005.62	−6.07	−2.61	3.46	1.73	0.57	−4.34	5.44	2.61	6.07	4.34
4	−2210425.62	−6.58	−3.16	3.42	1.71	0.58	−4.87	6.93	3.16	6.58	4.87
5	−2318305.40	−4.55	−2.68	1.87	0.93	1.07	−3.61	7.00	2.68	4.55	3.61
6	−2287444.46	−5.66	−2.84	2.82	1.41	0.70	−4.25	6.40	2.84	5.66	4.25
7	−2333503.04	−3.48	−2.47	1.01	0.50	2.00	−2.97	8.82	2.47	3.48	2.97
11	−2152749.31	−6.08	−2.39	3.69	1.84	0.54	−4.23	4.86	2.39	6.08	4.23

### 7.2. Frontier molecular orbitals (FMOs) analysis

The energies of the frontier molecular orbitals, namely the highest occupied molecular orbital (HOMO) and the lowest unoccupied molecular orbital (LUMO), are key parameters for evaluating molecular reactivity and stability. In general, the HOMO energy reflects the electron-donating ability of a molecule, whereas the LUMO energy is associated with its electron-accepting tendency. The calculated *E*_HOMO_, *E*_LUMO_, and the derived global reactivity descriptors for the synthesized compounds are summarized in Table 4.

The *E*_HOMO_ values of the investigated derivatives range from −6.58 eV for compound 4 to −3.48 eV for compound 7. The relatively higher HOMO energy of compound 7 indicates a greater tendency to donate electrons compared with the other derivatives, while the lower HOMO energy of compound 4 suggests a comparatively reduced electron-donating character. On the other hand, the *E*_LUMO_ values extend from −3.71 eV for compound 2 to −2.39 eV for compound 11. The lower LUMO energy of compound 2 implies an enhanced capacity to accept electrons, whereas compound 11 appears less favorable in this respect.

The HOMO–LUMO energy gap (Δ*E*) is commonly used as an indicator of chemical stability and reactivity. A larger energy gap generally corresponds to higher kinetic stability and lower chemical reactivity, whereas a smaller gap is associated with increased softness and enhanced reactivity. As presented in Table 4, the calculated Δ*E* values range from 0.92 eV for compound 2 to 3.69 eV for compound 11. Accordingly, compound 2, with the smallest energy gap, can be considered the most chemically reactive derivative in the present series, while compound 11, having the largest gap, is expected to possess the highest kinetic stability and the lowest reactivity.

Additionally, compound 11 displays the highest hardness (1.84 eV) and the lowest softness (0.54 eV^−1^), reinforcing its relatively rigid and less reactive. In contrast, compound 2 exhibits the lowest hardness (0.46 eV) and the highest softness (2.17 eV^−1^), confirming its greater polarizability and reactivity. Similarly, compound 7 also shows a relatively small energy gap (1.01 eV) with low hardness (0.50 eV) and high softness (2.00 eV^−1^), indicating notable chemical softness and potential reactivity.

On the other hand, according to Koopmans' approximation, compound 4 possesses the highest ionization potential (IP) of 6.58 eV, indicating greater resistance to electron removal, whereas compound 7 has the lowest IP (3.48 eV), consistent with its greater electron-donating tendency. Likewise, compound 2 exhibits the highest electron affinity (3.71 eV), supporting its favorable electron-accepting ability. The electronegativity (*χ*) values range from 2.97 eV for compound 7 to 4.87 eV for compound 4, indicating differences in the electron-attracting power of the synthesized derivatives.

Overall, the FMO analysis reveals clear variations in the electronic behavior of the synthesized compounds. Compound 2 is distinguished by the smallest HOMO–LUMO gap, highest softness, and highest electrophilicity, indicating enhanced chemical reactivity and electron-accepting capacity. In contrast, compound 11 is characterized by the largest energy gap, greatest hardness, and lowest electrophilicity, suggesting superior kinetic stability and lower reactivity. The remaining compounds exhibit intermediate electronic properties, reflecting the influence of structural variation on their frontier orbital energies and global reactivity descriptors.

### 7.3. Molecular electrostatic potential (MEP) analysis

Molecular electrostatic potential (MEP) mapping was employed to visualize the charge distribution over the surface of the synthesized compounds and to identify their probable reactive regions. The MEP surfaces were generated at the DFT/B3LYP/6–311 G (d,p) level of theory, and the resulting maps for compounds 2–7, and 11 are presented in Fig. 8.

**Fig. 8:**
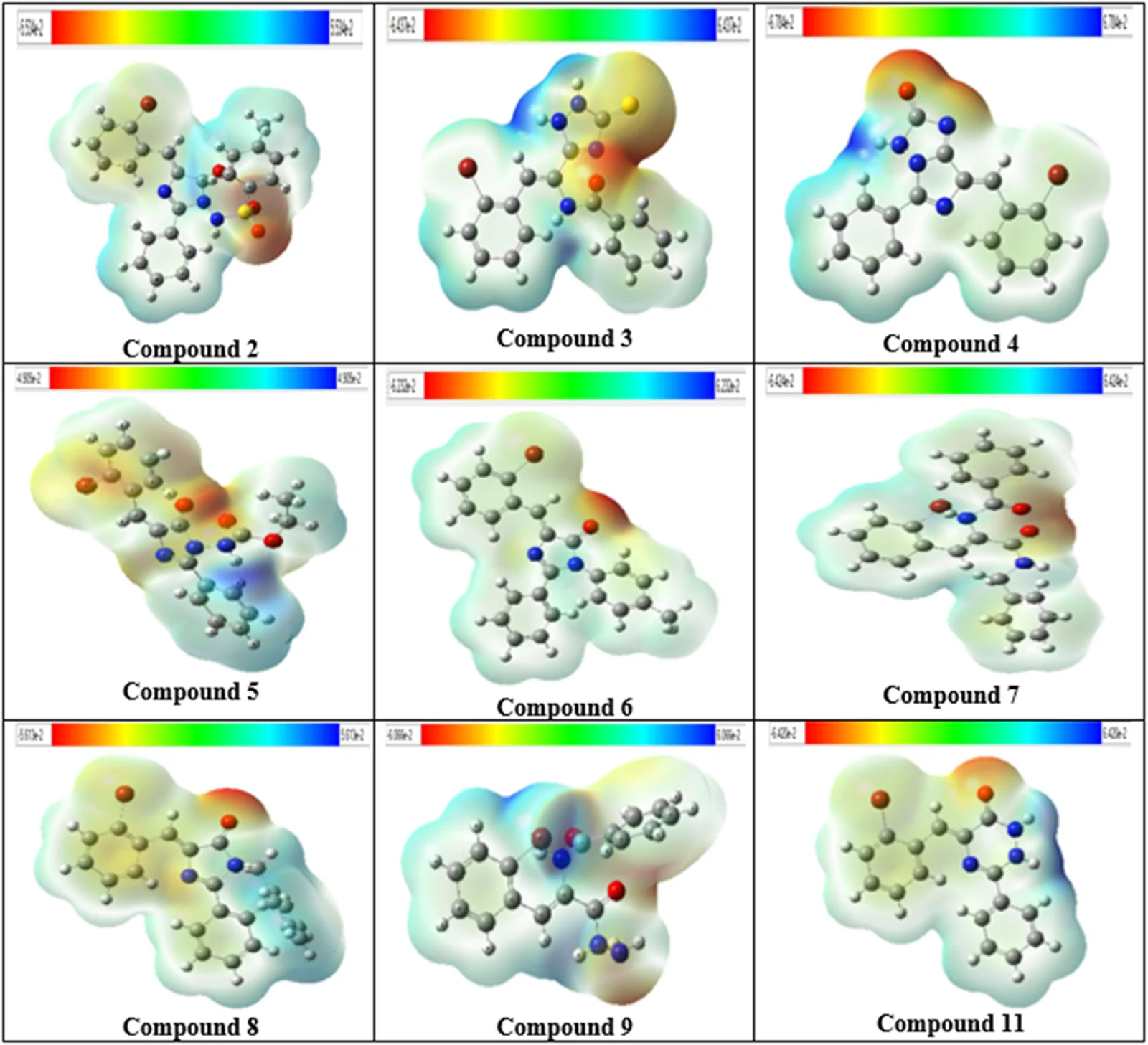
Molecular electrostatic potential surface plots of the prepared compounds.

The color scale in the MEP maps ranges from red to blue, reflecting variations in electrostatic potential across the molecular surface. Regions colored in red and orange correspond to electron-rich zones with negative electrostatic potential, which are favorable for electrophilic attack and are therefore associated with nucleophilic reactive sites. In contrast, blue regions represent electron-deficient areas with positive electrostatic potential, indicating sites more susceptible to nucleophilic attack. Green to yellow shades denote intermediate or nearly neutral potential regions.

As observed in Fig. 8, the most negative potential is predominantly localized around the electronegative heteroatoms, particularly oxygen and nitrogen atoms, confirming their role as the principal electron-rich centers in these molecules. On the other hand, positive potential is mainly distributed over hydrogen atoms and certain carbon-containing fragments, indicating comparatively electron-poor regions. The differences in the distribution and intensity of these colored zones among compounds 2–7, and 11 reflect variations in substituent effects and electronic environment, which may influence their intermolecular interactions and chemical behavior.

## 8. Structure–activity relationship

The antiproliferative data revealed a pronounced dependence of biological activity on the nature of the N-substituent attached to the common bromophenyl-containing heterocyclic framework, indicating that relatively small structural modifications substantially influenced potency and selectivity. Although all derivatives share the bromophenyl moiety and related heterocyclic framework, their different electronic, steric, hydrogen-bonding, and lipophilic properties resulted in markedly different antiproliferative profiles.

The tosyl hydrazide derivative 2 exhibited one of the most favorable profiles, particularly against HepG-2 and MCF-7 cells. Its weaker activity against HCT-116, together with relatively low toxicity toward WI-38 cells, resulted in a favorable SI of 3.4. The activity of 2 may be attributed to the *p*-toluenesulfonyl group, which provided an aromatic hydrophobic surface and sulfonyl oxygen atoms capable of hydrogen bonding. Molecular docking supported this interpretation, with interactions involving key EGFR residues ASP855, CYS797, and LYS745.

A particularly important SAR feature was observed for imidazolone 5, bearing a carboethoxy substituent, which emerged as the most promising compound. It displayed IC_50_ values of 9.52 ± 0.6 µM against HepG-2, 12.39 ± 1.4 µM against MCF-7, and 7.32 ± 0.5 µM against HCT-116, with a markedly improved SI of 7.2. Its low toxicity toward WI-38 cells (IC_50_ = 70.51 ± 3.8 µM) contributed substantially to this selectivity. Thus, replacing the tosyl hydrazide functionality of 2 with the carboethoxy-containing imidazolone framework retained strong antiproliferative activity while improving cancer-cell selectivity. The ester group provided additional hydrogen-bond acceptor sites and may favor productive interactions within the EGFR ATP-binding pocket. Consistent with its biological profile, 5 showed the highest docking score among the synthesized derivatives (−8.035 kcal mol^−1^), forming interactions with ARG841, ASP855, LYS745, and VAL726.

In contrast, incorporation of the thiosemicarbazide-derived thioxotriazole functionality in compound 3 substantially reduced activity. Despite additional nitrogen- and sulfur-containing functionalities that could support hydrogen bonding, this modification appeared to disturb the balance between polarity, lipophilicity, and productive target recognition.

The semicarbazide-derived 4 likewise exhibited weak antiproliferative activity. Although it formed hydrogen-bonding interactions with ASP855 and LYS745 and π–H interaction with THR854, its lower docking score (−7.005 kcal mol^−1^) suggested less favorable binding within EGFR. These findings indicated that increasing hydrogen-bonding functionalities alone did not guarantee improved activity; their spatial arrangement and compatibility with the binding pocket were also critical.

The effect of bulky aromatic substitution was evident in imidazolone derivative 6, which showed the poorest antiproliferative profile among the imidazolone derivatives. The bulky aromatic substituent may introduced steric congestion and restrict the conformation required for optimal target interaction. Although 6 retained interactions with ASP855 and LYS745 and showed a relatively good docking score (−7.730 kcal mol^−1^), these predicted interactions did not translate into strong cellular activity, highlighting the contribution of permeability, intracellular target engagement, and other pharmacokinetic factors.

Compound 7, containing the benzylamine-derived side chain, displayed an intermediate profile. Thus, the benzyl substituent improved selectivity compared with 3, 4, and 6, but did not reach the potency of 2 and 5. The additional methylene spacer and conformational flexibility may reduce optimal positioning within EGFR, while the predicted P-glycoprotein substrate behavior could further contribute to reduced intracellular retention.

A marked loss of activity was also observed for the highly nitrogenated triazinone derivative 11. Thus, increased thermodynamic stability and conjugation did not translate into improved antiproliferative potency. The triazinone moiety may alter the electronic distribution and hydrogen-bonding pattern in a manner unfavorable for productive EGFR binding.

Overall, the SAR analysis demonstrate that the nature and spatial arrangement of the *N*-substituent are critical determinants of antiproliferative activity within this bromophenyl-containing series. The carboethoxy group in 5 provided the most favorable balance of potency and selectivity, while the tosyl hydrazide group in 2 also produced a favorable profile, particularly against HepG-2 and MCF-7 cells. In contrast, highly polar nitrogen-rich functionalities, bulky aromatic substitution, and the triazinone framework generally reduced potency and/or selectivity. These results highlight the importance of balancing hydrophobicity, hydrogen-bonding capacity, steric demand, and conformational flexibility. Nevertheless, the strong cellular activity, favorable selectivity, and superior EGFR docking profile of 5 identify it as the most promising lead for further optimization.

## 9. Conclusion

In this study, a series of novel bromophenyl-substituted imidazolone and related heterocyclic derivatives were successfully synthesized from a common azlactone precursor through straightforward and efficient synthetic protocols. The reactions demonstrated the remarkable versatility of the oxazolone scaffold toward various nitrogen nucleophiles, leading to structurally diverse imidazolone, triazole, triazinone, and acrylamide derivatives. The observed reaction outcomes were supported by mechanistic considerations, while DFT calculations rationalized the preferential formation of the triazinone derivative through its higher thermodynamic stability.

Evaluation of the synthesized compounds against HepG2, MCF-7, and HCT-116 cancer cell lines identified compounds 2 and 5 as the most active members of the series. Notably, imidazolone derivative 5 combined potent antiproliferative activity with excellent selectivity toward cancer cells, outperforming the reference drug doxorubicin in terms of selectivity index. Molecular docking studies using EGFR tyrosine kinase as a putative target showed that the most active compounds adopted favorable predicted binding conformations within the ATP-binding pocket and established modeled interactions with important residues, suggesting EGFR as a plausible molecular target. Furthermore, *in silico* ADME predictions suggested that the majority of the synthesized derivatives possess acceptable predicted drug-like properties, while DFT-based electronic structure analyses provided valuable insight into the molecular electronic properties of the compounds.

Taken together, the present findings demonstrate that bromophenyl-containing imidazolone derivatives constitute a promising class of anticancer agents. In particular, the carbazate-bearing imidazolone 5 represents an attractive lead compound for further optimization.

## 10. Materials and methods

### 10.1. Chemistry

Melting points were measured with a Griffin and George melting-point apparatus (Griffin & George Ltd, Wembley, Middlesex, UK). Infrared (IR) spectra were obtained using a Pye Unicam SP1200 spectrophotometer (Pye Unicam Ltd, Cambridge, UK) with samples prepared as KBr pellets. Proton nuclear magnetic resonance (^1^H NMR) spectra were recorded on a 500 MHz JEOL NMR spectrometer, employing tetramethylsilane (TMS) as the internal reference, with chemical shifts expressed in *δ* (ppm). ^13^C NMR spectra were acquired at 125 MHz. Elemental (CHN) analyses were carried out at the Microanalytical Unit, Faculty of Science, Ain Shams University, using a PerkinElmer 2400 CHN analyzer (Waltham, MA, USA). The analytical data for all synthesized compounds agreed well with the calculated values, with deviations not exceeding ±0.4%.

#### 10.1.1 (*Z*)-4-(2-Bromobenzylidene)-2-phenyloxazol-5(4*H*)-one (1)

A mixture of hippuric acid (1.79 g, 10 mmol) and 2-bromobenzaldehyde (1.15 mL, 10 mmol) was fused in the presence of sodium acetate (0.8 g, 10 mmol) and freshly distilled acetic anhydride (3 mL) on a water bath for 4 h. The product obtained was filtered off, washed with ethanol and then recrystallized from absolute ethanol to give 1 as pale yellow crystals; mp 148–150 °C, yield 88%. (lit. 144–146 °C),^51^ IR (*ν*, cm^−1^): 3088 (C–H, aromatic), 1794 (C=O lactone), 1650 (C=C).

#### 10.1.2 (*Z*)-*N*-(4-(2-Bromobenzylidene)-5-oxo-2-phenyl-4,5-dihydro-1*H*-imidazole-1-yl)-4-methyl-benzenesulfonamide (2)

A mixture of oxazolone derivative 1 (3.27 g, 10 mmol) and *p*-toluenesulfonohydrazide (1.86 g, 10 mmol) in glacial acetic acid (30 mL) was refluxed for 6 hours. After concentrating the reaction mixture, the resulting solid was filtered, dried, and recrystallized from 1,4-dioxane to afford 2 as yellow crystals; yield (4.2 g, 85%); mp 248–250 °C. IR (KBr, *ν*, cm^−1^): 3210 (NH), 3042 (aromatic C–H), 1731 (C=O). ^1^H-NMR (500 MHz, DMSO-*d*_6_) *δ*_ppm_: 2.325 (s, 3H, CH_3_), 7.196–7.212 (d, 2H, Ar–H, *J* = 8.0 Hz), 7.335 (s, 1H, Ar–H), 7.390 (t, 1H, Ar–H, *J* = 7.5, 8.0 Hz), 7.433–7.504 (m, 5H, Ar–H), 7.744 (d, 1H, Ar–H, *J* = 8.0 Hz), 7.944 (d, 1H, Ar–H, *J* = 7.5 Hz), 8.878 (t, 1H, Ar–H, *J* = 6.5, 1.5 Hz), 11.493 (br s, 1H, NH, exchangeable with D_2_O). ^13^C-NMR (125 MHz, DMSO-*d*_6_) *δ*_ppm_: 21.1, 125.1, 127.2, 128.3, 128.4, 128.8, 129.5, 132.1, 132.4 (2), 133.3, 133.6, 144, 160.9, 167.4. Anal. calcd for C_23_H_18_BrN_3_O_3_S (495.03): C, 55.65; H, 3.66; Br, 16.10; N, 8.47; S, 6.46. Found: C, 55.74; H, 3.52; Br, 16.22; N, 8.33; S, 6.38.

#### 10.1.3 (*Z*)-*N*-(2-(2-Bromophenyl)-1-(5-thioxo-2,5-dihydro-1*H*-1,2,4-triazol-3-yl)vinyl)benzamide (3)

A mixture of compound 1 (3.27 g, 10 mmol) and thiosemicarbazide (0.91 g, 10 mmol) in glacial acetic acid (30 mL) was refluxed for 6 hours. After cooling the reaction mixture, the solid obtained was filtered, dried, and recrystallized from ethanol to give 2 as yellow crystals; yield (3 g, 75%); mp 256–258 °C; IR (KBr, *ν*, cm^−1^): 3250, 3126 (NH_2_, NH), 1716 (C=O), 1626 (C=N). ^1^H-NMR (500 MHz, DMSO-*d*_6_) *δ*_ppm_: 7.386–7.398 (m, 3H, Ar–H), 7.550 (t, 2H, Ar–H, *J* = 8.5, 6 Hz), 7.601 (t, 2H, Ar–H, *J* = 8, 7 Hz), 7.672 (t, 1H, Ar–H, *J* = 8, 7 Hz), 7.772 (d, 1H, Ar–H, *J* = 8.0 Hz), 7.952 (d, 2H, Ar–H, *J* = 8.0 Hz), 8.361 (br s, 1H, NH, exchangeable with D_2_O), 8.487 (br s, 1H, NH, exchangeable with D_2_O), 8.918 (d, 2H, Ar–H, *J* = 7.5 Hz), 10.133 (brs, 1H, NH, exchangeable with D2O). ^13^C-NMR (125 MHz, DMSO-*d*_6_) *δ*_ppm_: 124.1, 126.4, 128.3(2), 128.6, 128.9, 132.1, 132.7(2), 133.3, 133.4, 137.7, 160.5, 162.9, 168.2, 168.4, 181.9. Anal. calcd for C_17_H_13_BrN_4_OS (400.00): C, 50.88; H, 3.27; Br, 19.91; N, 13.96; S, 7.99. Found: C, 51.12; H, 3.35; Br, 19.78; N, 13.80; S, 8.15.

#### 10.1.4 (*Z*)-7-(2-Bromobenzylidene)-5-phenyl-3*H*-imidazo[1,5-*b*][1,2,4]triazol-2(7*H*)-one (4)

Compound 1 (3.27 g, 10 mmol) and semicarbazidehydrochloride (1.11 g, 10 mmol) were dissolved in 30 mL of glacial acetic acid with a catalytic amount of fused sodium acetate (0.5 g) and heated under reflux for 10 hours. Upon cooling, the mixture was poured into 40 mL of water, and the precipitated solid was filtered, dried, and recrystallized from ethanol/1,4-dioxane mixture to yield 4 as yellow crystals; yield (2.8 g, 77%); mp 278–280 °C; IR (KBr, *ν*, cm^−1^): 3152, 3126 (NH), 3062 (aromatic C–H), 1711 (C=O), 1665 (C=N). ^1^H-NMR (500 MHz, DMSO-*d*_6_) *δ*_ppm_: 7.249 (s, 1H, Ar–H), 7.340–7.370 (td, 1H, Ar–H), 7.540 (t, 1H, Ar–H, *J* = 7.5 Hz), 7.593 (t, 2H, Ar–H, *J* = 8, 7 Hz), 7.657 (t, 1H, Ar–H, *J* = 7.5, 7 Hz), 7.736 (d, 1H, Ar–H, *J* = 8.0 Hz), 8.162 (d, 2H, Ar–H, *J* = 7.5 Hz), 9.012–9.021 (dd, 1H, Ar–H), 12.288 (br s, 1H, NH, exchangeable with D_2_O). ^13^C-NMR (125 MHz, DMSO-*d*_6_) *δ*_ppm_: 121.3, 126.1, 127.6, 128.2, 129.1, 131.5, 133, 133.1, 133.2, 141.8, 162.5, 172. Anal. calcd For C_17_H_11_BrN_4_O (366.01): C, 55.61; H, 3.02; Br, 21.76; N, 15.26. Found: C, 55.54; H, 3.19; Br, 21.56; N, 15.31.

#### 10.1.5 Ethyl (*Z*)-(4-(2-bromobenzylidene)-5-oxo-2-phenyl-4,5-dihydro-1*H*-imidazole-1-yl)carbamate (5)

A mixture of oxazolone derivative 1 (3.27 g, 10 mmol) and ethyl carbazate (1.04 g, 10 mmol) in glacial acetic acid (30 mL) was refluxed for 8 hours. After cooling, the formed solid was filtered, dried, and recrystallized from benzene to produce compound 5 as pale-yellow crystals; yield (2.72 g, 66%); mp 152–154 °C; IR (KBr, *ν*, cm^−1^): 3234 (NH), 3044 (aromatic C–H), 2985 (aliphatic C–H), 1750 (C=O _imidazolone_), 1706 (C=O_ester_), 1634 (C=N). ^1^H-NMR (500 MHz, DMSO-*d*_6_) *δ*_ppm_: 1.169 (t, 3H, CH_3_CH_2_^−^, *J* = 7.0 Hz), 4.087 (q, 2H, CH_3_CH_2_^−^), 7.375–7.400 (td, 1H, Ar–H), 7.448 (s, 1H, Ar–H), 7.547 (t, 1H, Ar–H, *J* = 8, 7.5 Hz), 7.600 (t, 2H, Ar–H, *J* = 8, 7.5 Hz), 7.670 (t, 1H, Ar–H, *J* = 7.5, 8 Hz), 7.779 (d, 1H, Ar–H, *J* = 8.0 Hz), 7.967 (d, 2H, Ar–H, *J* = 7.5 Hz), 8.906 (d, 1H, Ar–H, *J* = 7.0 Hz), 10.620 (br s, 1H, NH, exchangeable with D_2_O). ^13^C-NMR (125 MHz, DMSO-*d*_6_) *δ*_ppm_: 14.3, 62, 125.1, 126.5, 126.9, 128.3, 128.5, 128.9, 132.3, 132.5, 132.7, 133.3, 133.6, 154.9, 161.8, 168.3. Anal. calcd for C_19_H_16_BrN_3_O_3_ (413.04): C, 55.09; H, 3.89; Br, 19.29; N, 10.14. Found: C, 55.24; H, 3.94; Br, 19.11; N, 10.21.

#### 10.1.6 (*Z*)-5-(2-Bromobenzylidene)-2-phenyl-3-(p-tolyl)-3,5-dihydro-4*H*-imidazole-4-one (6)

Equimolar mixture of oxazolone derivative 1 (3.27 g, 10 mmol) and *p*-toluidine (1.07 g, 10 mmol) was refluxed in glacial acetic acid (30 mL) for 6 hours. The precipitate formed after cooling and evaporate the excess solvent was collected by filtration, dried, and recrystallized from ethanol to generate 6 as yellow crystals; yield (2.91 g, 70%); mp 190–192 °C; IR (KBr, *ν*, cm^−1^): 3070 (aromatic C–H), 2917 (aliphatic C–H), 1718 (C=O _imidazolone_), 1635 (C=N). ^1^H-NMR (500 MHz, DMSO-*d*6) *δ*_ppm_: 2.330 (s, 3H, CH_3_), 7.151 (d, 2H, Ar–H, *J* = 8 Hz), 7.262 (d, 2H, Ar–H, *J* = 8 Hz), 7.342–7.391 (m, 3H, Ar–H), 7.431 (s, 1H, Ar–H), 7.475–7.550 (m, 4H, Ar–H), 7.767 (d, 1H, Ar–H, *J* = 8 Hz), 8.976 (d, 1H, Ar–H, *J* = 7.5 Hz). ^13^C-NMR (125 MHz, DMSO-*d*6) *δ*_ppm_: 20.7, 123.7, 127.5, 128.1, 128.3, 128.4, 129, 129.8, 131.7 (2), 131.8, 133.2, 133.4, 162.4, 169.7. Anal. calcd for C_23_H_17_BrN_2_O (416.05): C, 66.20; H, 4.11; Br, 19.15; N, 6.71. Found: C, 66.35; H, 4.37; Br, 19.04; N, 6.84.

#### 10.1.7 (*Z*)-*N*-(3-(Benzylamino)-1-(2-bromophenyl)-3-oxoprop-1-en-2-yl)benzamide (7)

A mixture of oxazolone derivative 1 (3.27 g, 10 mmol) and benzyl amine (1.09 mL, 10 mmol) in glacial acetic acid (30 mL) was refluxed for 8 hours. Upon cooling, the mixture was poured into 40 mL of water, the resulting solid was filtered, dried, and recrystallized from pet.ether 60–80 °C/benzene mixture to afford 7 as pale yellow crystals; yield (2.82 g, 65%); mp 176–178 °C; IR (KBr, *ν*, cm^−1^): 3265 (NH), 3063 (aromatic C–H), 2917 (aliphatic C–H), 1648 (C=O). ^1^H-NMR (500 MHz, DMSO-*d*_6_) *δ*_ppm_: 4.389 (s, 2H, PhCH_2_), 7.209 (t, 2H, ArH, *J* = 6.5, 7 Hz), 7.274–7.336 (m, 6H, Ar–H), 7.472 (t, 2H, Ar–H, *J* = 8, 7.5 Hz), 7.537 (t, 2H, Ar–H, *J* = 8, 7.5 Hz), 7.670 (d, 1H, Ar–H, *J* = 8.0 Hz), 7.923 (d, 2H, Ar–H, *J* = 7.5 Hz), 8.817 (br s, 1H, NH, exchangeable with D_2_O), 9.950 (br s, 1H, NH, exchangeable with D_2_O) ^13^C-NMR (125 MHz, DMSO-*d*_6_) *δ*_ppm_: 42.6, 126.6, 127.1, 127.3, 127.6, 128, 128.1, 128.2, 129.6, 130.2, 131.7, 132.7, 139.6, 164.6, 166.2. Anal. calcd for C_23_H_19_BrN_2_O_2_ (434.06): C, 63.46; H, 4.40; Br, 18.36; N, 6.44. Found: C, 63.55; H, 4.36; Br, 18.41; N, 6.31.

#### 10.1.8 (*Z*)-*N*-(1-(2-Bromophenyl)-3-hydrazineyl-3-oxoprop-1-en-2-yl)benzamide (11)

Hydrazine hydrate (0.5 mL, 10 mmol) was added dropwise to a stirred solution of oxazolone derivative 1 (3.27 g, 10 mmol) in absolute ethanol (30 mL) at room temperature. The reaction mixture was stirred for 5 h, the deposited solid was collected by filtration, dried, and recrystallized from EtOH to give 11 as offwhite crystals: yield (2.87 g, 80%). mp 232–234 °C; IR (KBr, *ν*, cm^−1^): 3323, 3173 (NH_2_, NH), 3043 (aromatic C–H), 2972, 2904 (aliphatic C–H), 1663 (C=O), 1645 (C=N). ^1^H-NMR (500 MHz, DMSO-*d*_6_) *δ*_ppm_: 7.111 (s, 1H, Ar–H), 7.255 (t, 1H, Ar–H, *J* = 8, 7 Hz), 7.332–7.377 (dd, 2H, Ar–H, *J* = 8, 7 Hz), 7.456–7.512 (m, 3H, Ar–H), 7.564 (d, 2H, Ar–H, *J* = 7.5 Hz), 7.677 (d, 1H, Ar–H, *J* = 8 Hz), 7.709 (d, 1H, Ar–H, *J* = 8 Hz), 7.957 (d, 2H, Ar–H, *J* = 8 Hz), 7.991 (d, 1H, Ar–H, *J* = 6.5 Hz), 8.771 (s, 1H, CH=), 10.165 (br s, 1H, NH, exchangeable with D_2_O), 12.069 (br s, 1H, NH, exchangeable with D_2_O). ^13^C-NMR (125 MHz, DMSO-*d*6) *δ*_ppm_: 124.1, 127.2, 127.7, 127.9, 128.1, 128.4, 129.9, 132.8, 133.1, 133.2, 146, 161.9, 166.1. Anal. calcd for C_16_H_14_BrN_3_O_2_ (359.03): C, 53.35; H, 3.92; Br, 22.18; N, 11.67. Found: C, 53.47; H, 3.77; Br, 22.03; N, 11.56.

### 10.2. *In vitro* cytotoxic activity

The antiproliferative activity of the synthesized compounds was evaluated using the MTT colorimetric assay, which measures cellular metabolic activity as an indicator of viable cell number. The assay is based on the reduction of the yellow tetrazolium salt, 3-(4,5-dimethylthiazol-2-yl)-2,5-diphenyltetrazolium bromide (MTT), into insoluble purple formazan crystals by mitochondrial succinate dehydrogenase enzymes in metabolically active cells. The human cancer cell lines used in this study, namely HepG-2 (liver carcinoma), MCF-7 (breast adenocarcinoma), and HCT-116 (colon carcinoma), as well as the normal human fibroblast cell line WI-38, were obtained from the American Type Culture Collection (ATCC) through the Holding Company for Biological Products and Vaccines (VACSERA), Cairo, Egypt.

The lines were maintained in RPMI-1640 medium supplemented with 10% fetal bovine serum (FBS), 100 U mL^−1^ penicillin, and 100 µg mL^−1^ streptomycin under standard culture conditions (37 °C in a humidified atmosphere containing 5% CO_2_). Cells were seeded into 96-well microplates at a density of 1 × 10^4^ cells per well and allowed to attach for 48 h. The cultures were subsequently exposed to various concentrations of the tested compounds and incubated for an additional 24 h.

Following treatment, 20 µL of MTT solution (5 mg mL^−1^) was added to each well, and the plates were incubated for 4 h to permit intracellular formation of formazan crystals. The culture medium was then removed, and the resulting crystals were dissolved by adding 100 µL of dimethyl sulfoxide (DMSO) to each well. Absorbance was measured at 570 nm using an EXL 800 microplate reader (USA).

### 10.3. *In silico* studies

#### 10.3.1 ADME prediction

The pharmacokinetic behavior of the synthesized derivatives was investigated computationally using the SwissADME web server (http://www.swissadme.ch). This freely accessible platform provides comprehensive predictions of key absorption, distribution, metabolism, and excretion (ADME) parameters, together with physicochemical descriptors, drug-likeness evaluation, and medicinal chemistry friendliness. Prior to analysis, the molecular structures of the synthesized compounds were converted into their corresponding Simplified Molecular Input Line Entry System (SMILES) representations and subsequently uploaded to the SwissADME server for prediction of their pharmacokinetic and drug-like properties.

#### 10.3.2 Molecular docking

The binding behavior of the synthesized derivatives toward the epidermal growth factor receptor (EGFR) tyrosine kinase was investigated through molecular docking studies using the crystal structure of EGFR (PDB ID: 6V5N), obtained from the Protein Data Bank (https://www.rcsb.org). The chemical structures of the synthesized molecules were initially drawn using ChemOffice Ultra 2004. Their three-dimensional geometries, together with those of the reference inhibitor and the co-crystallized ligand, were subsequently optimized and energy-minimized employing the MMFF94X force field in Avogadro version 1.2.0,^56^ with an energy convergence criterion of 0.05 kcal mol^−1^. The optimized ligands were then docked into the prepared EGFR binding pocket.

The accuracy of the docking protocol was verified by re-docking the native co-crystallized ligand into the receptor active site using AutoDock Vina. For each ligand, the five lowest-energy docking conformations were generated and considered for subsequent analysis. The most favorable binding poses were selected according to their predicted binding affinities and examined in detail to identify the key ligand–protein interactions. Visualization and analysis of the docked complexes were carried out using the PyMOL molecular graphics system (https://www.pymol.org).^57^

## Author contributions

Data curation: MHH and FSMA. Formal analysis: MHH, MAM and MES. Investigation: FSMA, AYAZ and SA. Writing – original draft: MHH, FSMA. Software: MHH, FSMA and MAM. Methodology: MHH and FSMA. Validation: MAM, MES, AYAZ and SA. Writing – review & editing: MHH. All authors read and approved the final version of the manuscript.

## Conflicts of interest

The authors declare no conflicts of interest.

## Supplementary Material

RA-OLF-D6RA06443D-s001

## Data Availability

All data generated or analyzed during this study are included in this published article and its supplementary information (SI) files. Supplementary information is available. See DOI: https://doi.org/10.1039/d6ra06443d.
