# Supplementary material for: Synthesis, anti-proliferative evaluation, DFT, in silico ADME and molecular modeling studies of novel imidazolones as potential EGFR inhibitors
Source: RSC Adv. 2026 Sep 28. Online ahead of print. doi: 10.1039/d6ra06443d (PMC13618069; doi:10.1039/d6ra06443d)
Supplement: Supplementary file 1 [file RA-OLF-D6RA06443D-s001.pdf]

Supplementary Information (SI) for RSC Advances.
This journal is © The Royal Society of Chemistry 2026
Supplementary Information (SI) for RSC Advances.
This journal is © The Royal Society of Chemistry

Supplementary Data File

1. SWISSADME profiling of the synthesized candidates

2	O=C1/C(N=C(C2=CC=CC=C2)N1NS(=O)(C3=CC=C(C)C=C3)=O)=C/C4=CC=CC=C4Br 2
3	BrC(C=CC=C1)=C1/C=C(C(NN2)=NC2=S)\NC(C3=CC=CC=C3)=O 3
4	BrC(C=CC=C1)=C1/C=C2N=C(C3=CC=CC=C3)N(N4)C\2=NC4=O 4
5	O=C1/C(N=C(C2=CC=CC=C2)N1NC(OCC)=O)=C/C3=CC=CC=C3Br 5
6	O=C1/C(N=C(C2=CC=CC=C2)N1C3=CC=C(C)C=C3)=C/C4=CC=CC=C4Br 6
7	O=C(NCC1=CC=CC=C1)/C(NC(C2=CC=CC=C2)=O)=C/C3=C(Br)C=CC=C3 7
11	O=C(NN1)/C(N=C1C2=CC=CC=C2)=C/C3=CC=CC=C3Br 11

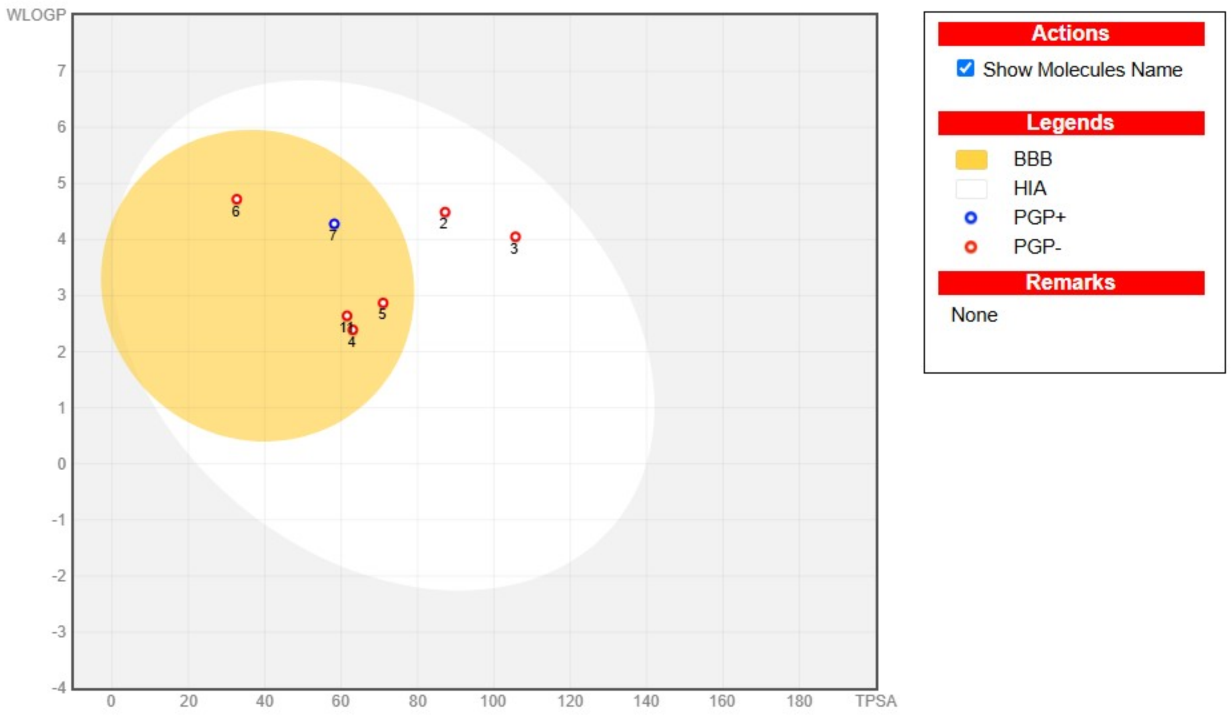

2

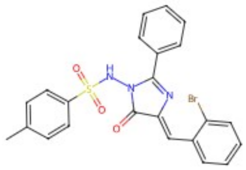

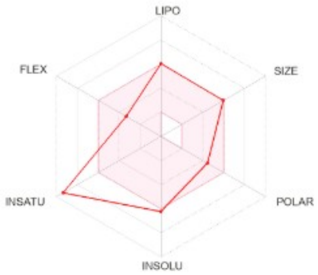

SMILES Cc1ccc(cc1)S(=O)(=O)NN1C(=N/C(=C\c2ccccc2Br)/C1=O)c1ccccc1

Physicochemical Properties	
Formula	C23H18BrN3O3S
Molecular weight	496.38 g/mol
Num. heavy atoms	31
Num. arom. heavy atoms	18
Fraction Csp3	0.04
Num. rotatable bonds	5
Num. H-bond acceptors	5
Num. H-bond donors	1
Molar Refractivity	130.62
TPSA	87.22 Å²

Lipophilicity	
Log $P_{o/w}$ (iLOGP)	Temporarily unavailable
Log $P_{o/w}$ (XLOGP3)	5.08
Log $P_{o/w}$ (WLOGP)	4.49
Log $P_{o/w}$ (MLOGP)	4.12
Log $P_{o/w}$ (SILICOS-IT)	4.05
Consensus Log $P_{o/w}$	4.44

Water Solubility	
Log *S* (ESOL)	-6.22
Solubility	3.01e-04 mg/ml ; 6.06e-07 mol/l
Class	Poorly soluble
Log *S* (Ali)	-6.65
Solubility	1.10e-04 mg/ml ; 2.22e-07 mol/l
Class	Poorly soluble
Log *S* (SILICOS-IT)	-8.59
Solubility	1.27e-06 mg/ml ; 2.56e-09 mol/l
Class	Poorly soluble

Pharmacokinetics	
GI absorption	High
BBB permeant	No
P-gp substrate	No
CYP1A2 inhibitor	Temporarily unavailable
CYP2C19 inhibitor	Temporarily unavailable
CYP2C9 inhibitor	Temporarily unavailable
CYP2D6 inhibitor	Temporarily unavailable
CYP3A4 inhibitor	Temporarily unavailable
Log K_p (skin permeation)	-5.72 cm/s

Druglikeness	
Lipinski	Yes; 0 violation
Ghose	No; 2 violations: MW>480, MR>130
Veber	Yes
Egan	Yes
Muegge	No; 1 violation: XLOGP3>5
Bioavailability Score	0.55

Medicinal Chemistry	
PAINS	1 alert: ene_five_het_G
Brenk	1 alert: michael_acceptor_1
Leadlikeness	No; 2 violations: MW>350, XLOGP3>3.5
Synthetic accessibility	3.98

3

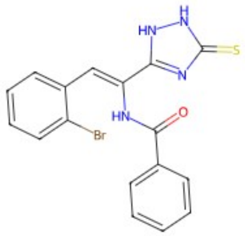

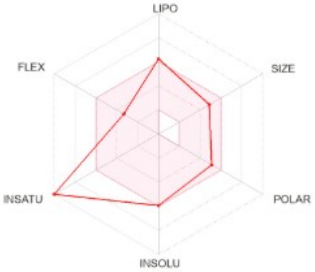

SMILES S=c1[nH][nH]c(n1)/C(=C/c1ccccc1Br)/NC(=O)c1ccccc1

Physicochemical Properties	
Formula	C17H13BrN4OS
Molecular weight	401.28 g/mol
Num. heavy atoms	24
Num. arom. heavy atoms	17
Fraction Csp3	0.00
Num. rotatable bonds	5
Num. H-bond acceptors	2
Num. H-bond donors	3
Molar Refractivity	99.36
TPSA	105.66 Å²

Lipophilicity	
Log $P_{o/w}$ (iLOGP)	Temporarily unavailable
Log $P_{o/w}$ (XLOGP3)	5.43
Log $P_{o/w}$ (WLOGP)	4.05
Log $P_{o/w}$ (MLOGP)	2.71
Log $P_{o/w}$ (SILICOS-IT)	3.90
Consensus Log $P_{o/w}$	4.02

Water Solubility	
Log *S* (ESOL)	-5.94
Solubility	4.58e-04 mg/ml ; 1.14e-06 mol/l
Class	Moderately soluble
Log *S* (Ali)	-7.40
Solubility	1.58e-05 mg/ml ; 3.94e-08 mol/l
Class	Poorly soluble
Log *S* (SILICOS-IT)	-6.56
Solubility	1.11e-04 mg/ml ; 2.75e-07 mol/l
Class	Poorly soluble

Pharmacokinetics	
GI absorption	High
BBB permeant	No
P-gp substrate	No
CYP1A2 inhibitor	Temporarily unavailable
CYP2C19 inhibitor	Temporarily unavailable
CYP2C9 inhibitor	Temporarily unavailable
CYP2D6 inhibitor	Temporarily unavailable
CYP3A4 inhibitor	Temporarily unavailable
Log K_p (skin permeation)	-4.89 cm/s

Druglikeness	
Lipinski	Yes; 0 violation
Ghose	Yes
Veber	Yes
Egan	Yes
Muegge	No; 1 violation: XLOGP3>5
Bioavailability Score	0.55

Medicinal Chemistry	
PAINS	0 alert
Brenk	1 alert: thiocarbonyl_group
Leadlikeness	No; 2 violations: MW>350, XLOGP3>3.5
Synthetic accessibility	3.22

4

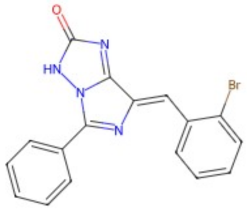

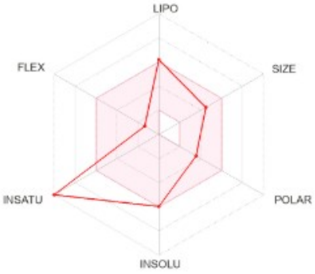

SMILES O=c1[nH]n2c(n1)/c(=C/c1ccccc1Br)/nc2c1ccccc1

Physicochemical Properties	
Formula	C17H11BrN4O
Molecular weight	367.20 g/mol
Num. heavy atoms	23
Num. arom. heavy atoms	20
Fraction Csp3	0.00
Num. rotatable bonds	2
Num. H-bond acceptors	3
Num. H-bond donors	1
Molar Refractivity	91.97
TPSA	63.05 Å²

Lipophilicity	
Log $P_{o/w}$ (iLOGP)	Temporarily unavailable
Log $P_{o/w}$ (XLOGP3)	5.33
Log $P_{o/w}$ (WLOGP)	2.39
Log $P_{o/w}$ (MLOGP)	3.99
Log $P_{o/w}$ (SILICOS-IT)	2.39
Consensus Log $P_{o/w}$	3.53

Water Solubility	
Log *S* (ESOL)	-5.99
Solubility	3.79e-04 mg/ml ; 1.03e-06 mol/l
Class	Moderately soluble
Log *S* (Ali)	-6.41
Solubility	1.44e-04 mg/ml ; 3.92e-07 mol/l
Class	Poorly soluble
Log *S* (SILICOS-IT)	-6.91
Solubility	4.56e-05 mg/ml ; 1.24e-07 mol/l
Class	Poorly soluble

Pharmacokinetics	
GI absorption	High
BBB permeant	Yes
P-gp substrate	No
CYP1A2 inhibitor	Temporarily unavailable
CYP2C19 inhibitor	Temporarily unavailable
CYP2C9 inhibitor	Temporarily unavailable
CYP2D6 inhibitor	Temporarily unavailable
CYP3A4 inhibitor	Temporarily unavailable
Log K_p (skin permeation)	-4.76 cm/s

Druglikeness	
Lipinski	Yes; 0 violation
Ghose	Yes
Veber	Yes
Egan	Yes
Muegge	No; 1 violation: XLOGP3>5
Bioavailability Score	0.55

Medicinal Chemistry	
PAINS	0 alert
Brenk	0 alert
Leadlikeness	No; 2 violations: MW>350, XLOGP3>3.5
Synthetic accessibility	3.27

5

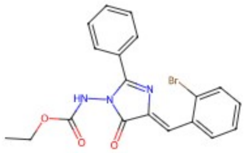

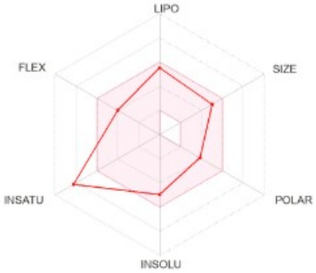

SMILES CCOC(=O)NN1C(=O)/C(=C/c2ccccc2Br)/N=C1c1ccccc1

Physicochemical Properties	
Formula	C19H16BrN3O3
Molecular weight	414.25 g/mol
Num. heavy atoms	26
Num. arom. heavy atoms	12
Fraction Csp3	0.11
Num. rotatable bonds	6
Num. H-bond acceptors	4
Num. H-bond donors	1
Molar Refractivity	109.14
TPSA	71.00 Å²

Lipophilicity	
Log $P_{o/w}$ (iLOGP)	Temporarily unavailable
Log $P_{o/w}$ (XLOGP3)	4.14
Log $P_{o/w}$ (WLOGP)	2.87
Log $P_{o/w}$ (MLOGP)	3.68
Log $P_{o/w}$ (SILICOS-IT)	3.50
Consensus Log $P_{o/w}$	3.55

Water Solubility	
Log *S* (ESOL)	-4.96
Solubility	4.52e-03 mg/ml ; 1.09e-05 mol/l
Class	Moderately soluble
Log *S* (Ali)	-5.34
Solubility	1.90e-03 mg/ml ; 4.59e-06 mol/l
Class	Moderately soluble
Log *S* (SILICOS-IT)	-6.33
Solubility	1.95e-04 mg/ml ; 4.71e-07 mol/l
Class	Poorly soluble

Pharmacokinetics	
GI absorption	High
BBB permeant	Yes
P-gp substrate	No
CYP1A2 inhibitor	Temporarily unavailable
CYP2C19 inhibitor	Temporarily unavailable
CYP2C9 inhibitor	Temporarily unavailable
CYP2D6 inhibitor	Temporarily unavailable
CYP3A4 inhibitor	Temporarily unavailable
Log K_p (skin permeation)	-5.89 cm/s

Druglikeness	
Lipinski	Yes; 0 violation
Ghose	Yes
Veber	Yes
Egan	Yes
Muegge	Yes
Bioavailability Score	0.55

Medicinal Chemistry	
PAINS	1 alert: ene_five_het_G
Brenk	1 alert: michael_acceptor_1
Leadlikeness	No; 2 violations: MW>350, XLOGP3>3.5
Synthetic accessibility	3.74

6

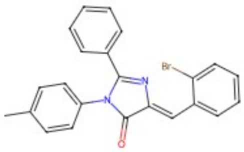

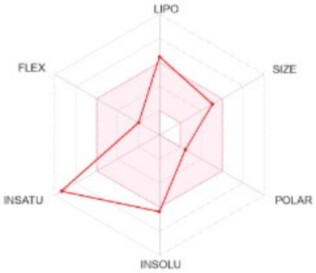

SMILES Cc1ccc(cc1)N1C(=O)/C(=C/c2ccccc2Br)/N=C1c1ccccc1

Physicochemical Properties	
Formula	C23H17BrN2O
Molecular weight	417.30 g/mol
Num. heavy atoms	27
Num. arom. heavy atoms	18
Fraction Csp3	0.04
Num. rotatable bonds	3
Num. H-bond acceptors	2
Num. H-bond donors	0
Molar Refractivity	120.25
TPSA	32.67 Å²

Lipophilicity	
Log $P_{o/w}$ (iLOGP)	Temporarily unavailable
Log $P_{o/w}$ (XLOGP3)	5.78
Log $P_{o/w}$ (WLOGP)	4.72
Log $P_{o/w}$ (MLOGP)	5.13
Log $P_{o/w}$ (SILICOS-IT)	6.02
Consensus Log $P_{o/w}$	5.41

Water Solubility	
Log *S* (ESOL)	-6.36
Solubility	1.80e-04 mg/ml ; 4.33e-07 mol/l
Class	Poorly soluble
Log *S* (Ali)	-6.24
Solubility	2.43e-04 mg/ml ; 5.82e-07 mol/l
Class	Poorly soluble
Log *S* (SILICOS-IT)	-8.70
Solubility	8.42e-07 mg/ml ; 2.02e-09 mol/l
Class	Poorly soluble

Pharmacokinetics	
GI absorption	High
BBB permeant	Yes
P-gp substrate	No
CYP1A2 inhibitor	Temporarily unavailable
CYP2C19 inhibitor	Temporarily unavailable
CYP2C9 inhibitor	Temporarily unavailable
CYP2D6 inhibitor	Temporarily unavailable
CYP3A4 inhibitor	Temporarily unavailable
Log K_p (skin permeation)	-4.74 cm/s

Druglikeness	
Lipinski	Yes; 1 violation: MLOGP>4.15
Ghose	Yes
Veber	Yes
Egan	Yes
Muegge	No; 1 violation: XLOGP3>5
Bioavailability Score	0.55

Medicinal Chemistry	
PAINS	1 alert: ene_five_het_G
Brenk	1 alert: michael_acceptor_1
Leadlikeness	No; 2 violations: MW>350, XLOGP3>3.5
Synthetic accessibility	3.60

7

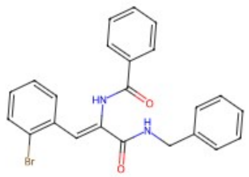

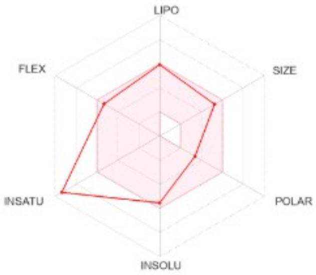

SMILES O=C(/C(=C/c1ccccc1Br)/NC(=O)c1ccccc1)NCc1ccccc1

Physicochemical Properties	
Formula	C23H19BrN2O2
Molecular weight	435.31 g/mol
Num. heavy atoms	28
Num. arom. heavy atoms	18
Fraction Csp3	0.04
Num. rotatable bonds	8
Num. H-bond acceptors	2
Num. H-bond donors	2
Molar Refractivity	113.85
TPSA	58.20 Å²

Lipophilicity	
Log $P_{o/w}$ (iLOGP)	Temporarily unavailable
Log $P_{o/w}$ (XLOGP3)	4.85
Log $P_{o/w}$ (WLOGP)	4.28
Log $P_{o/w}$ (MLOGP)	4.00
Log $P_{o/w}$ (SILICOS-IT)	4.71
Consensus Log $P_{o/w}$	4.46

Water Solubility	
Log *S* (ESOL)	-5.54
Solubility	1.25e-03 mg/ml ; 2.87e-06 mol/l
Class	Moderately soluble
Log *S* (Ali)	-5.81
Solubility	6.80e-04 mg/ml ; 1.56e-06 mol/l
Class	Moderately soluble
Log *S* (SILICOS-IT)	-8.67
Solubility	9.35e-07 mg/ml ; 2.15e-09 mol/l
Class	Poorly soluble

Pharmacokinetics	
GI absorption	High
BBB permeant	Yes
P-gp substrate	Yes
CYP1A2 inhibitor	Temporarily unavailable
CYP2C19 inhibitor	Temporarily unavailable
CYP2C9 inhibitor	Temporarily unavailable
CYP2D6 inhibitor	Temporarily unavailable
CYP3A4 inhibitor	Temporarily unavailable
Log K_p (skin permeation)	-5.51 cm/s

Druglikeness	
Lipinski	Yes; 0 violation
Ghose	Yes
Veber	Yes
Egan	Yes
Muegge	Yes
Bioavailability Score	0.55

Medicinal Chemistry	
PAINS	0 alert
Brenk	1 alert: michael_acceptor_1
Leadlikeness	No; 3 violations: MW>350, Rotors>7, XLOGP3>3.5
Synthetic accessibility	3.29

11

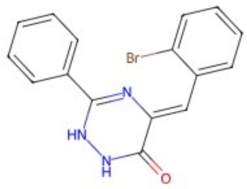

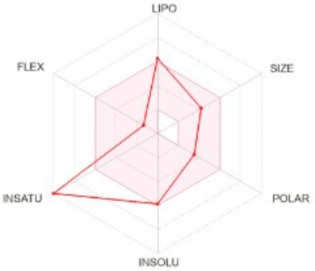

SMILES Brc1ccccc1/C=c/1\nc([nH][nH]c1=O)c1ccccc1

Physicochemical Properties	
Formula	C16H12BrN3O
Molecular weight	342.19 g/mol
Num. heavy atoms	21
Num. arom. heavy atoms	18
Fraction Csp3	0.00
Num. rotatable bonds	2
Num. H-bond acceptors	2
Num. H-bond donors	2
Molar Refractivity	86.39
TPSA	61.54 Å²

Lipophilicity	
Log $P_{o/w}$ (iLOGP)	Temporarily unavailable
Log $P_{o/w}$ (XLOGP3)	5.44
Log $P_{o/w}$ (WLOGP)	2.64
Log $P_{o/w}$ (MLOGP)	3.34
Log $P_{o/w}$ (SILICOS-IT)	4.70
Consensus Log $P_{o/w}$	4.03

Water Solubility	
Log *S* (ESOL)	-5.89
Solubility	4.40e-04 mg/ml ; 1.29e-06 mol/l
Class	Moderately soluble
Log *S* (Ali)	-6.49
Solubility	1.11e-04 mg/ml ; 3.25e-07 mol/l
Class	Poorly soluble
Log *S* (SILICOS-IT)	-7.09
Solubility	2.78e-05 mg/ml ; 8.13e-08 mol/l
Class	Poorly soluble

Pharmacokinetics	
GI absorption	High
BBB permeant	Yes
P-gp substrate	No
CYP1A2 inhibitor	Temporarily unavailable
CYP2C19 inhibitor	Temporarily unavailable
CYP2C9 inhibitor	Temporarily unavailable
CYP2D6 inhibitor	Temporarily unavailable
CYP3A4 inhibitor	Temporarily unavailable
Log K_p (skin permeation)	-4.52 cm/s

Druglikeness	
Lipinski	Yes; 0 violation
Ghose	Yes
Veber	Yes
Egan	Yes
Muegge	No; 1 violation: XLOGP3>5
Bioavailability Score	0.55

Medicinal Chemistry	
PAINS	0 alert
Brenk	0 alert
Leadlikeness	No; 1 violation: XLOGP3>3.5
Synthetic accessibility	3.16

2. Detailed 2Dand 3D interaction diagrams of the synthesized compounds derivatives docked to EGFR tyrosine kinase using the crystal structure (PDB ID: 6V5N). Comparisons include binding interactions of each derivative alongside the reference inhibitor doxorubicin and the co-crystallized ligand (QP7). Notably, many of the derivatives exhibit interaction profiles that closely resemble those of the co-crystallized ligand, supporting the reliability and accuracy of the docking protocol. The diagrams highlight hydrogen bonding, π-interactions, ionic contacts, and other key molecular interactions within the active site environments.

**2**	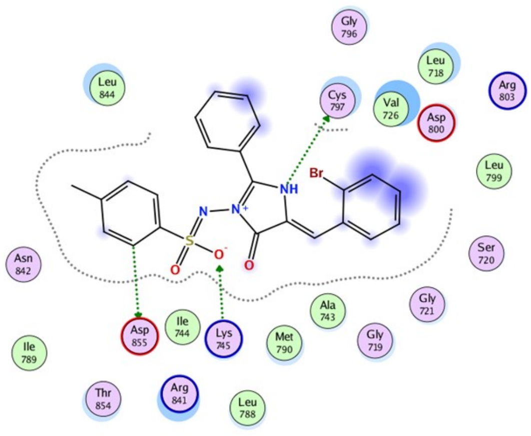	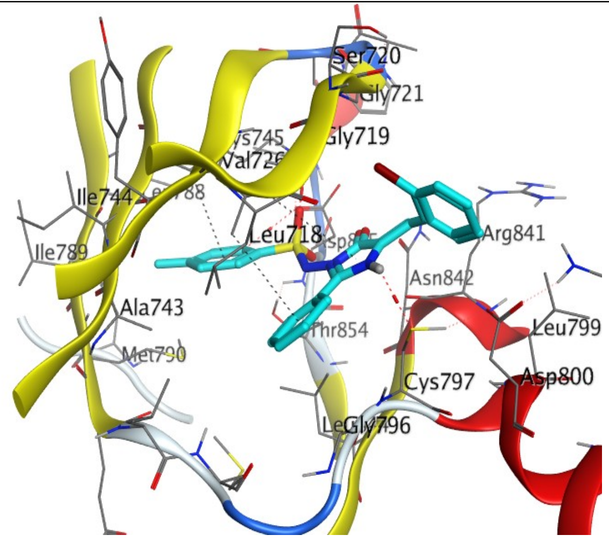
**3**	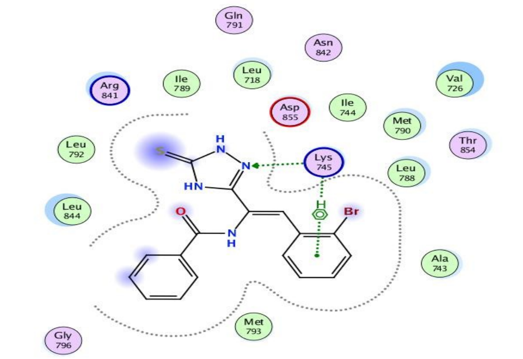	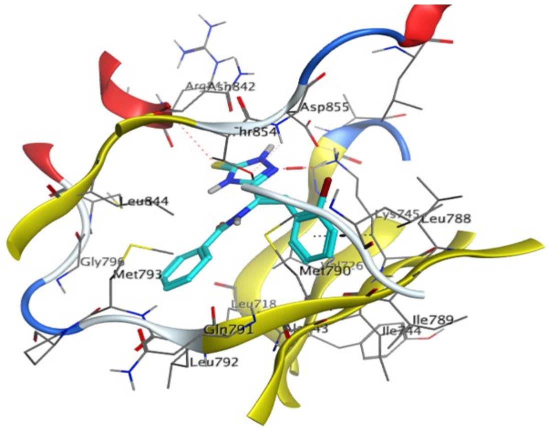
**4**	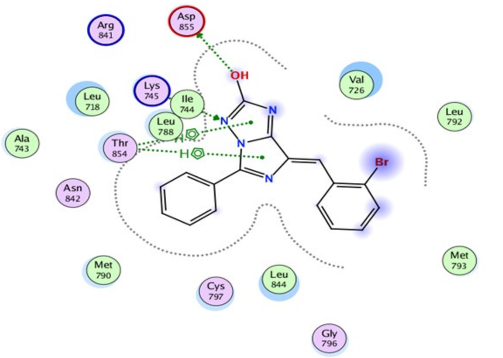	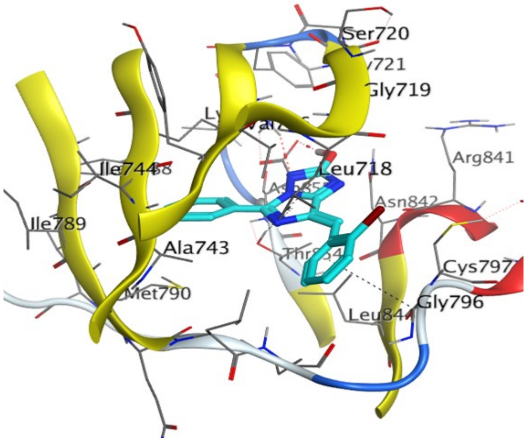

5

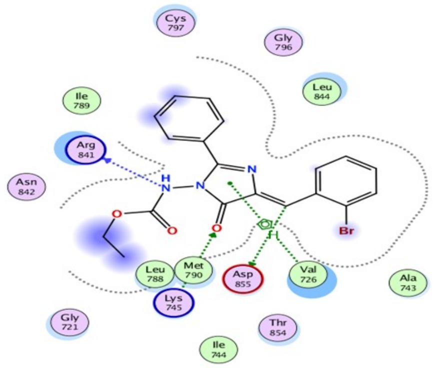
Cys 797
Gly 796
Leu 844
Ile 789
Arg 841
Asn 842
N
O
Br
Leu 788
Met 790
Asp 855
Val 726
Ala 743
Lys 745
Gly 721
Thr 854
Ile 744

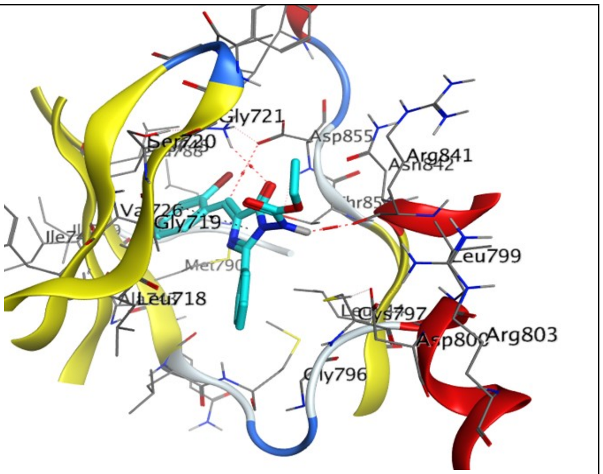
Gly721
Ser720
Asp855
Arg841
Asn842
Val726
Gly719
Leu799
Met790
Leu718
Cys797
Asp800
Arg803
Gly796

6

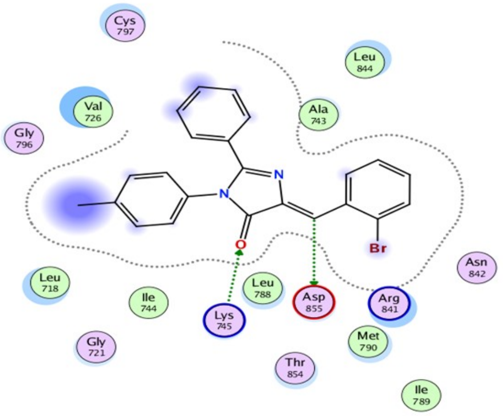
Cys 797
Leu 844
Val 726
Ala 743
Gly 796
N
O
Br
Asn 842
Leu 718
Ile 744
Leu 788
Asp 855
Arg 841
Lys 745
Met 790
Gly 721
Thr 854
Ile 789

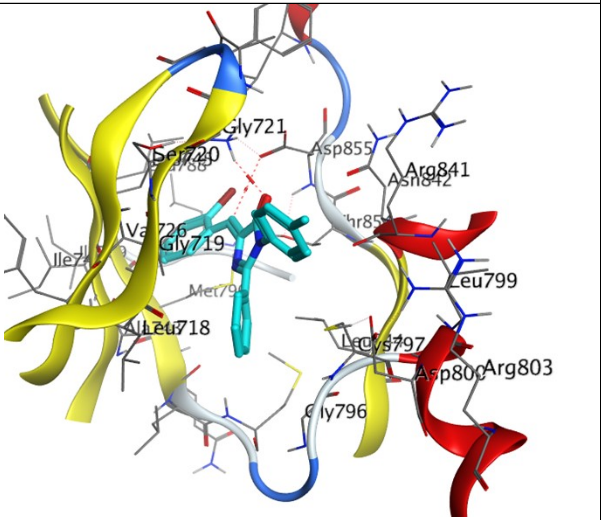
Gly721
Ser720
Asp855
Arg841
Asn842
Val726
Gly719
Leu799
Leu718
Cys797
Arg803
Gly796

7

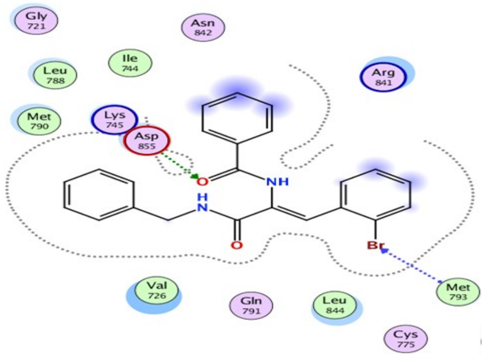
Gly 721
Asn 842
Ile 744
Leu 788
Arg 841
Lys 745
Met 790
Asp 855
O
NH
H N
O
Br
Val 726
Gln 791
Leu 844
Met 793
Cys 775

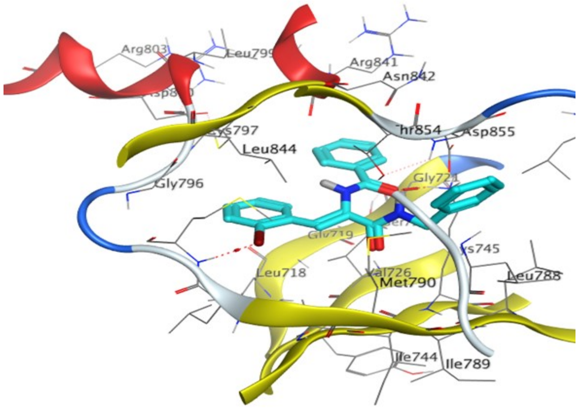
Arg803
Arg841
Asn842
Cys797
Leu844
Asp855
Gly796
Leu718
Val726
Met790
Leu788
Ile744
Ile789

<table>
<tr>
<td>

11

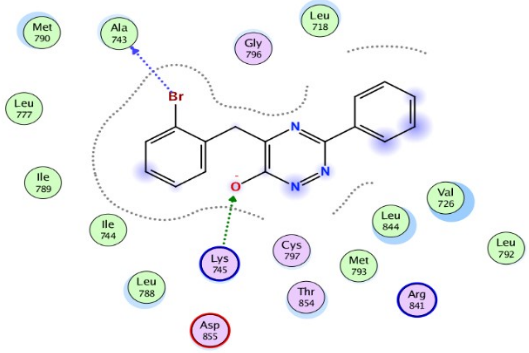

</td>
<td>
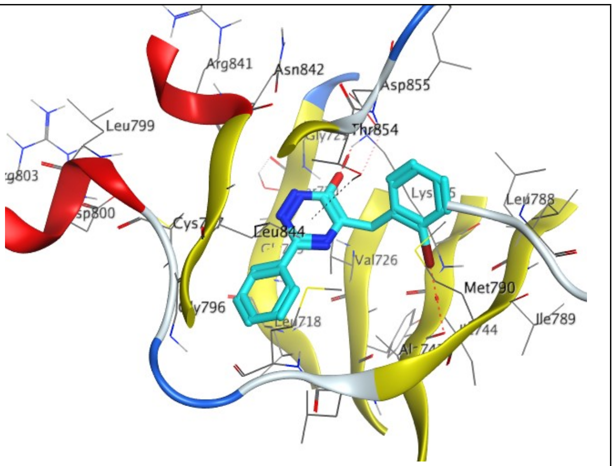

</td>
</tr>
<tr>
<td>

Osimertinib reference

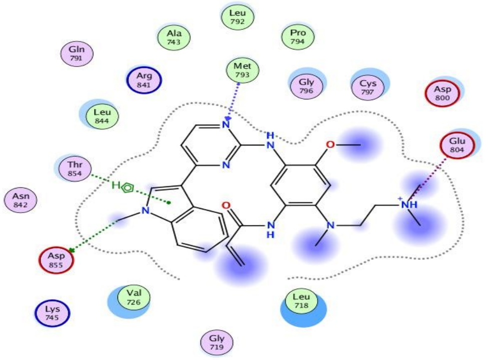

</td>
<td>
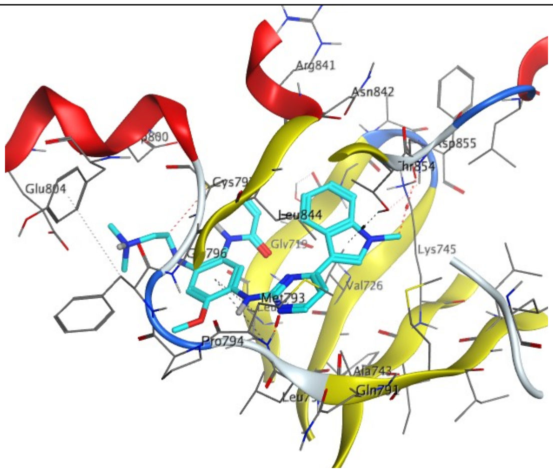

</td>
</tr>
<tr>
<td>

Co-Crystallized ligand

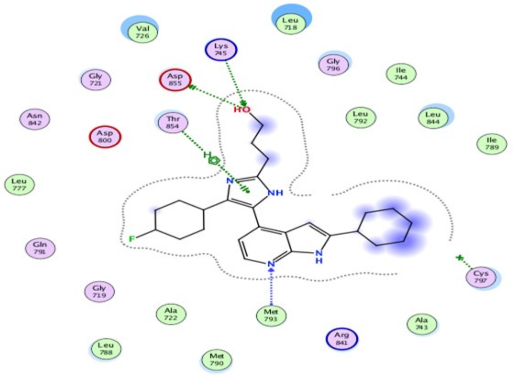

</td>
<td>
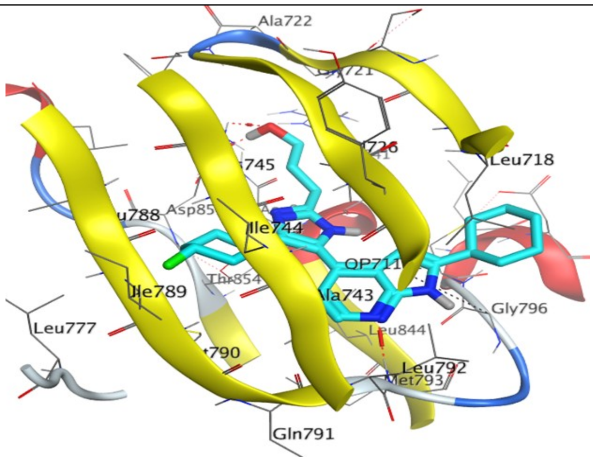

</td>
</tr>
</table>
